# Catalytic and Photocatalytic Electrospun Nanofibers for Hydrogen Generation from Ammonia Borane Complex: A Review

**DOI:** 10.3390/polym13142290

**Published:** 2021-07-13

**Authors:** Ahmed Abutaleb

**Affiliations:** Chemical Engineering Department, College of Engineering, Jazan University, Gizan 45142, Saudi Arabia; azabutaleb@jazanu.edu.sa or engahmedabutaleb@gmail.com

**Keywords:** electrospinning, catalysts, ceramic, C NFs, TiO_2_ NFs, ammonia borane, hydrogen generation

## Abstract

Hydrogen (H_2_) is a promising renewable energy source that can replace fossil fuels since it can solve several environmental and economic issues. However, the widespread usage of H_2_ is constrained by its storage and safety issues. Many researchers consider solid materials with an excellent capacity for H_2_ storage and generation as the solution for most H_2_-related issues. Among solid materials, ammonia borane (abbreviated hereafter as AB) is considered one of the best hydrogen storage materials due to its extraordinary H_2_ content and small density. However, the process must be conducted in the presence of efficient catalysts to obtain a reasonable amount of generated H_2_. Electrospun nanofibrous catalysts are a new class of efficient catalysts that involves the usage of polymers. Here, a comprehensive review of the ceramic-supported electrospun NF catalysts for AB hydrolysis is presented, with a special focus on catalytic and photolytic performance and preparation steps. Photocatalytic AB hydrolysis was discussed in detail due to its importance and promising results. AB photocatalytic hydrolysis mechanisms under light were also explained. Electrospun catalysts show excellent activity for AB hydrolysis with good recyclability. Kinetics studies show that the AB hydrolysis reaction is independent of AB concentration and the first-order reaction of NF catalysts.

## 1. Introduction

### 1.1. The Importance of Hydrogen

Due to the negative environmental impact and the limitations of fossil fuels, many countries are putting considerable efforts into the scientific research and development of renewable energy. Many researchers have deemed hydrogen (H_2_) as ideal and having the potential to be a promising renewable fuel [1,2,3].

H_2_ has many excellent characteristics such as a high energy density, availability, and cleanness; it is green and pollution-free since water is the main byproduct of the combustion reaction. However, the transportation and storage of H_2_ hinder its commercial applications due to its low density and high explosiveness. Recent research has identified H_2_ storage as a bottleneck of the widespread use of H_2_ energy [4,5,6]. H_2_ can be stored in high-pressure and cryogenic liquid storage tanks. Nevertheless, it is complicated to store H_2_ using these tanks because it has a very low boiling point (−252.87 °C) and very low gas density (0.08988 g/L) at 1 atm [7]. The weight of the storage containers and the possibility of developing leaks are the main factors that limit storing H_2_ in high-pressure tanks. To store H_2_ as a liquid, a refrigeration unit is needed to preserve the cryogenic state, which adds weight and energy costs and causes around 40% of energy loss. Furthermore, liquid or gaseous H_2_ storage poses significant safety issues for on-board transport applications [7]. Solid materials with high H_2_ storage and generation capacity are promising materials to overcome storage and transportation limitations. H_2_ storage materials should have a small volume, small mass, and high hydrogen content. These solid materials encounter a phase change by the appropriate process (heating, reaction, etc.) to produce hydrogen gas. Among all the storage materials, special attention has been paid recently to boron hydrides due to their unique properties that satisfy the requirements of the Department of Energy (DOE), USA [8]. Ammonia borane (NH_3_BH_3_, referred to hereafter as AB) is the most promising boron hydride chemical for H_2_ production.

### 1.2. Ammonia Borane (AB) as a Valuable Source of H_2_

AB is a solid chemical with a high H_2_ capacity (19.6 wt%) that is higher than the US Department of Energy’s target weight (5.5 wt%) [9]. Furthermore, it has a low density (0.74 g/cm^3^), low molecular weight (30.7 g/mol), high environmental safety, and great aqueous and alcohol stability. AB is highly stable in aqueous solutions and can remain so for more than 4 days. In the presence of an efficient catalyst, liquid AB can produce H_2_ gas (solid to liquid to gas phase change) at room temperature. Thus, AB has gained more attention over other H_2_ storage materials.

AB is formed from the electronegativity difference between boron (B) and nitrogen (N). Electronegative N takes electrons from its three H neighbors, leaving a partial negative charge. On the other hand, electropositive B gives electrons away to its three H neighbors, leaving a partial positive charge. The attraction forces between the opposite charges on the H or dihydrogen atoms hold solid AB together. On average, six dihydrogen bonds exist on each AB molecule; the bond between N and B (N–B) is a donor-acceptor bond that is weak compared to the covalent or ionic bond. Hence, this bond is easily broken by electron attacks [10].

Primarily, there are two different ways to release H_2_ from AB, namely pyrolysis and hydrolysis [11,12]. Pyrolysis requires a very high temperature; hence, it is not favorable from both the economic and safety points of view. Furthermore, the process generates undesirable byproducts. AB hydrolysis is the most promising way to generate H_2_ from AB [7,13,14,15,16]. Since 2006, the hydrolysis reaction has received substantial consideration, with special emphasis on catalytic materials [17]. AB can produce three equivalent moles of H_2_ gas by the hydrolytic decomposition reaction in the presence of a suitable catalyst according to the following reaction [14]:

NH_3_BH_3_ + 2 H_2_O → NH_4_BO_2_ + 3 H_2_ (1)

An efficient and low-cost catalyst is needed to catalyze the AB hydrolysis process under moderate conditions for it to be useful in real applications. Noble metals such as platinum (Pt), palladium (Pd), ruthenium (Ru), and rhodium (Rh) have been used to speed up the reaction due to their exceptional activity, stability, and durability. However, their high cost limits their practical applications. Researchers are putting significant effort into the fabrication of effective catalysts for AB hydrolysis using low-cost and highly available first-row transition metals such as cobalt (Co), nickel (Ni), copper (Co), and iron (Fe) [6,18,19].

Nanomaterials are defined, according to the National Science Foundation (NSF), as materials with at least one dimension less than 100 nm [20]. Nanomaterials are classified based on the number of dimensions outside the nanoscale range. Therefore, materials with all dimensions less than 100 nm are (0D) nanomaterials. The most common example of (0D) nanomaterial is nanoparticles. (1D) nanomaterials are materials with only one dimension bigger than 100 nm. Nanowires, nanotubes, nanorods, and nanofibers (NFs) are some examples of (1D) nanomaterials. (2D) nanomaterials are materials with two dimensions outside the nanoscale (only one dimension less than 100 nm). Plate-like-shape materials such as nanofilms and nanolayers are usually (2D) nanomaterials. Materials with all three dimensions outside the nanoscale range are called (3D) nanomaterials. (3D) nanomaterials include multinanowires and dispersion nanoparticles [21,22,23,24,25].

The form of the catalyst—for example, nanoparticles (NPs), nanorods (NRDs), or nanofibers (NFs)—plays an important role in catalytic performance. For instance, good catalytic activities can be achieved using NPs made from noble or transition metals. Unfortunately, the catalytic activities of most catalytic NPs decrease with time due to their agglomeration. Supporting catalytic NPs in different supportive forms has been demonstrated to overcome the previous challenges. Different techniques such as wet impregnation, chemical vapor deposition, and electrospinning (ES) have been applied to support NPs in different supports. ES has been utilized recently to make supported and nonsupported efficient nanofibrous catalysts [14].

## 2. Electrospun Fibrous Catalysts

### 2.1. Characteristics of Electrospun Catalysts

**Figure 1:**
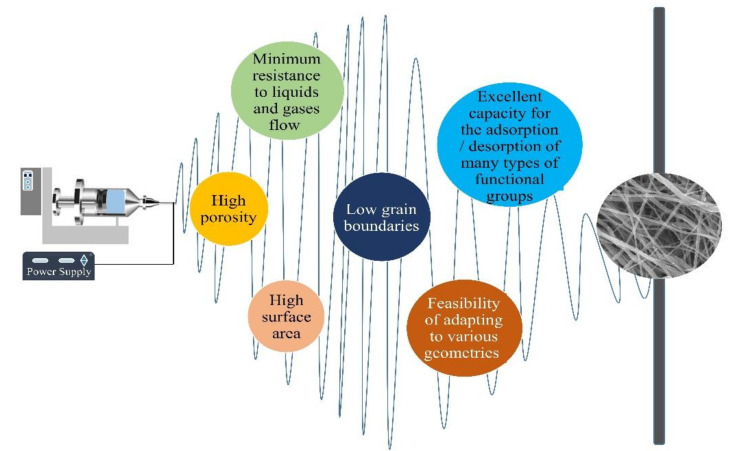
Properties of electrospun NFs.

Electrospun NFs have many outstanding characteristics that make them promising catalytic materials. Some of the properties of electrospun NFs are shown in Figure 1. Contrary to NPs, a mesh of fibers is self-supporting, and its high porosity and other characteristics provide several reaction sites. The stable structure of the nanofibrous membrane ensures a consistent and predictable reaction rate and poses minimum issues of the agglomeration of the catalytic material [10].

### 2.2. Electrospinning Technique

**Figure 2:**
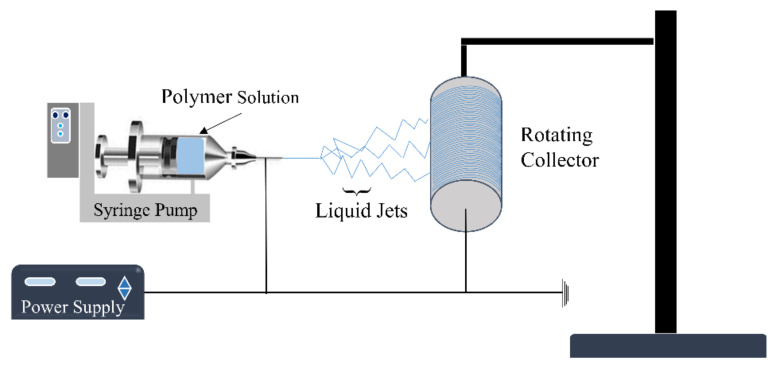
Schematic of electrospinning setup.

Historically, several methods have been applied to produce fibers of different dimensions. These methods include drawing, self-assembly [26,27], phase separation [27,28], template synthesis [27], and electrospinning (ES) [9,29,30,31,32,33,34,35]. Each production technique has its merits and demerits, and ES has been acknowledged as one of the most effective techniques to produce polymeric and ceramic fibers [9,31,35,36,37,38]. ES is cost-effective and can fabricate fibers continuously. This process fabricates fibers with controlled dimensions and desired structures. The diameter of the produced fibers can be achieved on the nanometer scale. The ES device generally comprises a reservoir of polymer dispersion solution with a pump, high-voltage source, nozzle, and conductive collector, as shown in Figure 2.

Organic and inorganic NFs can be fabricated using electrospinning. Polymers are involved in the fabrication of both types either as the raw material, in the case of organic NFs, or as a fabrication support, in the case of inorganic NFs.

In fact, wide ranges of natural and synthetic polymers have been electrospun to fabricate polymeric NFs for various applications. Polymers such as polyvinylpyrrolidone (PVP), polyacrylontrrile (PAN), poly(vinylacetate) (PVAc), polyvibyl alcohol (PVA), polyimide (PI), polyethylene oxide (PEO), polyvinylodene fluoride (PVDF), polystyrene (PS), and polyethersulfone (PES) are the most common electrospun polymers [39,40,41].

Polymers are also used to facilitate the fabrication of ceramic electrospun NFs. Ceramic precursor solutions do not possess sufficient viscosity to make a jet during electrospinning. Therefore, polymers are added to the electrospinning ceramic solutions to increase the viscosity to ensure successful electrospinning.

Sol–gel method, which includes a polymerization stage, is usually used in the fabrication of ceramic NFs via electrospinning. The electrospinning solution is prepared by adding ceramic precursors to a viscous polymer solution [42]. The metallic precursor and polymer mixture have to be completely miscible to form the gel network. Electrospinning of the sol–gel polymer solution can form either:

i. Single phase ceramic NFs obtained by removing the polymer NFs via heat treatment procedure;
ii. Polymer/ceramic hybrid NFs obtained without any heat treatment.

It is very important to note that the choice of the polymer, ceramic precursor, solvent, and additives, such as surfactants and salts, greatly affects the final properties of the electrospun polymer and ceramic NFs. For example, to prepare inorganic NFs, it is recommended to use metal acetates as a metallic precursor. Metal acetates are able to participate with the proper polymers in the polycondensation reactions to produce electrospinable sol–gels.

Based on the electrostatic field between the nozzle and collector, the electrospun fibers are collected on the surface of the collector screen. Many parameters influence ES operations and the characteristics of the produced fibers, including the type and nature of the collector, the voltage applied, the distance between the nozzle and collector, and the dispersion flow rate.

Electrospun fibers have extensive applications in industries such as air filtration, water purification [43,44], fabrication of sensors and biosensors [45], medical and biomedical applications (e.g., soft-tissue engineering, encapsulation of bioactive species, regenerative medicine, drug delivery fuel, and cell membranes) [45,46], antibiotics [47,48,49,50], environmental protection [51], smart textiles, surface coatings, and energy harvesting [33,52], conversion, catalysis [34,53], and storage, among others. Several valuable reviews have been published for the utilization of electrospun NFs in different application areas; a list of some of them is provided in Table 1. Nonetheless, there are no recent comprehensive reviews on the applications of electrospun ceramic NFs in catalysis for H2 production from AB yet. In this paper, an effort has been made to develop such a comprehensive review of the published literature on the applications of electrospun NFs as AB hydrolysis catalysts. It worth mentioning that the studies by Ayman et al. and Nasser et al. have contributed the most in the search for effective electrospun catalysts for AB hydrolysis [13,54].

## 3. Fabrication of Catalytic Electrospun Nanofibers for AB Hydrolysis

Ceramic-supported and nonsupported catalysts are the most famous and widely applied industrial catalysts. Ceramic catalysts have the advantage of tolerance against harsh reaction conditions. They can also be utilized as photocatalysts due to their activity under light. ES has been utilized successfully to fabricate ceramic NFs for various applications, including catalysis.

To prepare ceramic electrospun NFs, the process usually involves several major steps, such as the following:

- Preparation of the sol–gel solution by mixing a polymeric solution with a metal oxide precursor;
- ES of the sol–gel solution to make nanofibrous mats;
- Calcination of the fibrous mats at high temperatures to remove the polymer and convert the precursor to the desired ceramic form.

It is very important to note that calcination and reduction steps depend greatly on the desired catalysts. For example, calcination is usually operated in an air atmosphere if the desired catalysts should have metal oxides.

**Figure 3:**
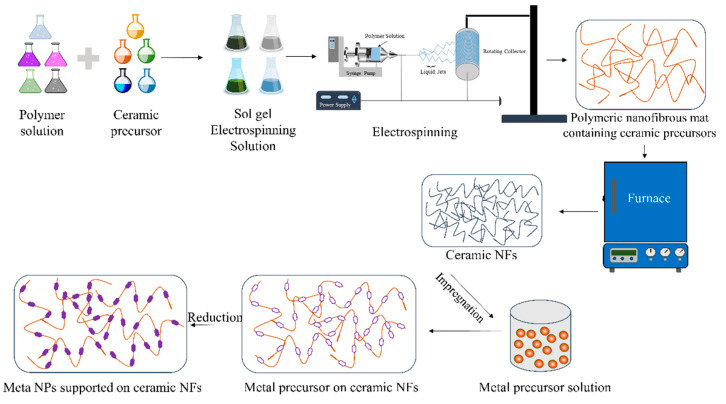
Preparation steps of supported ceramic nanofibrous catalysts using electrospinning.

The ceramic NFs can be utilized directly as catalysts or catalytic supports, depending on the reaction. To support catalytic metals or metal oxide NPs on the surface of ceramic NFs, the particles can be added to the sol–gel solution before ES, as shown in Figure 3. This technique uses the ES method as the primary method to fabricate the ceramic-supported catalysts. The metal precursors must be soluble in the sol–gel solution to obtain well-dispersed catalysts. A strong attachment between the metal NPs and the ceramic NFs is usually obtained using this fabrication method. However, it is sometimes challenging to dissolve metal precursors in the ES solutions.

**Figure 4:**
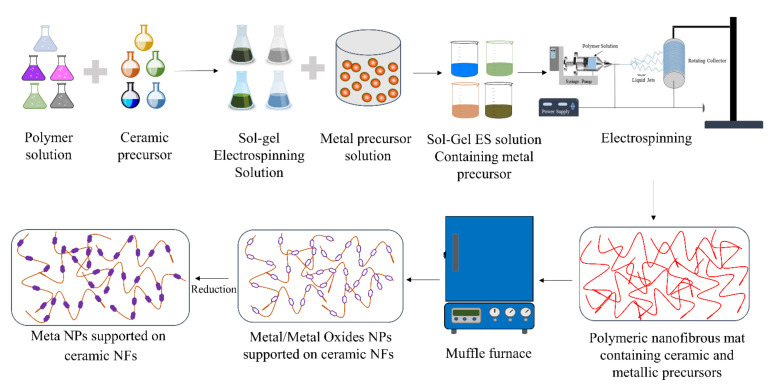
Preparation steps of supported ceramic nanofibrous catalysts using electrospinning followed by wet impregnation.

ES, followed by wet impregnation, chemical reduction, or any other catalytic fabrication technique, can also be used to make ceramic NF-supported catalysts, as shown in Figure 4. Here, ceramic NFs are first prepared using ES. Furthermore, metal NPs are dispersed on the fabricated ceramic NFs. This process makes it easy to disperse catalytic NPs on any ceramic NFs. However, the attachment of the NPs with the ceramic NFs is usually weaker than the catalysts that are made using electrospinning only, as described in Figure 3. In both methods, the reduction step is not essential and is usually performed if it is desired to convert metal precursor NPs to their pure metallic forms. It can be conducted with any reducing agent such as H_2_ or liquid hydrazine.

**Figure 5:**
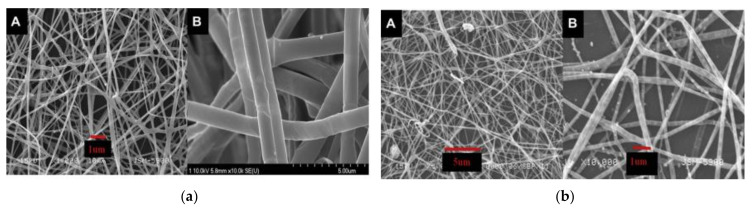
(**a**) (A) SEM; and (B) FE-SEM images of copper acetate (CuAc)/nickel acetate (NiAc)/PVA mat without heat treatment; (**b**) FE-SEM image of NiAC/CuAC/PVA mat NFs after sintering at 750 °C for 3 h in argon atmosphere. Reprinted from reference [54] with permission from Elsevier.

Heat treatment is a necessary step in the fabrication of ceramic NFs to remove the polymer and convert the ceramic raw material to the desired ceramic structure. The fibrous mat is usually heated in the presence of air, argon, nitrogen, or any other gases depending on the desired final structure of the ceramic NFs. As shown in Figure 5a,b, heat treatment decreased the size of the NFs since the polymer is degraded and the ceramic precursor is converted to a ceramic structure. It is very important to make sure that the NFs retain the nanofibrous form after heat treatment [54].

## 4. Reaction Setup for Testing Catalytic Performance toward AB Hydrolysis

**Figure 6:**
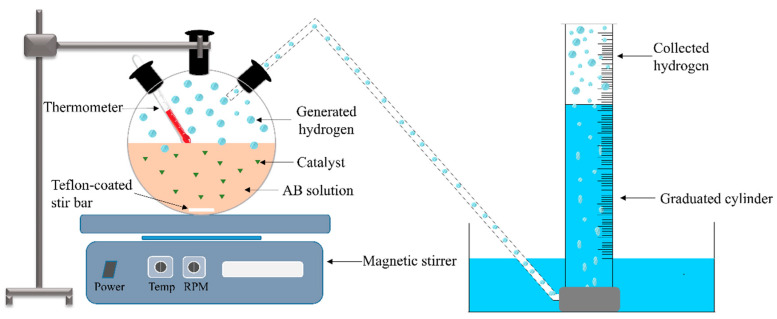
Schematic of batch reactor for testing the performance of electrospun catalysts for AB hydrolysis.

After the preparation of the NF catalysts, the catalytic performance toward AB hydrolysis is usually tested in a batch reactor, as shown in Figure 6. The traditional water displacement method is usually applied to measure the amount of H_2_ gas. Generally, a conventional three-necked round-bottomed flask is used as a reactor. The flask is placed in a magnetic stirrer to provide heat and stirring. The generated H_2_ from the reaction is channeled through tubing into a water-filled inverted measuring cylinder. The volume of the displaced water is equal to the generated H_2_ volume.

The reaction usually starts with the addition of the catalyst to the reaction mixture and ceases when no H_2_ gas is observed. It is very important to consider the amount of water vapor when interpreting the experimental results. The process can be considered as a multiphase process with phase changes where it involves liquid (AB in water), solid (electrospun catalyst), and gas (generated H_2_).

## 5. Nanofibrous Ceramic Catalysts for AB Hydrolysis

Many electrospun-supported and nonsupported ceramic catalysts have been prepared previously. The fabricated ceramic catalysts were tested in the absence or presence of light as normal catalysts and photocatalysts, respectively. Here, the catalysts are divided into three main categories, namely carbon NF (C NF)-supported catalysts, TiO_2_ NF-supported catalysts, and cobalt catalysts.

## 6. Carbon NFs as a Catalytic Support for AB Hydrolysis Reaction

C NFs are desirable materials for many applications including catalysis since they are chemically inert with valuable electrical and catalytic characteristics [90,91,92]. C NFs have been used as a catalytic support due to their large capacity for AB adsorption. In addition, C NFs assist in dispersing and stabilizing the active metals. Table 2 shows the catalytic data of C NF-supported catalysts for AB hydrolysis.

### 6.1. NiCu Nanorods on Carbon NFs

Yousef et al. have prepared a nonprecious nickel–copper (NiCu) nanorod-doped C NF (NiCu NRs/C NFs) catalyst for AB hydrolysis [54]. NiCu nanoalloy catalysts have been widely studied in various reactions such as fuel cell reactions. The nanoalloys have high catalytic and electrochemical catalytic activities [93,94].

According to the empirical Hume-Rothery rules, an alloy is formed if the atomic radii, valence, and electronegativity of the elements and crystal structures of the two metals are similar. The Ni and Cu alloy perfectly fulfills the Hume-Rothery rules since they have similar atomic radii (size mismatch∼2%), the same valence (+1), similar electronegativity (difference∼2%), and the same crystal structure (FCC).

It is very important to note that using the nanothermodynamics theory, the NiCu alloy cannot be obtained at room temperature, and substantial heat treatment is required to form the alloy [95].

i. To prepare NiCu NRs/C NFs, first, nickel acetate (NiAc) and copper acetate (CuAc) aqueous solutions were prepared. The aqueous solutions were mixed with a 10 wt% polyvinylpyrrolidone (PVP) aqueous solution. The sol–gel solution was mixed for 5 h at 50 °C to obtain a homogenous solution. The ES of the sol–gel solution was then conducted at 20 °C. The fabricated NFs were vacuum dried for a day at 60 °C. To remove the polymer, the NFs were calcined at 750 °C (2.3 °C/min) for 3 h in an argon (Ar) atmosphere.

**Figure 7:**
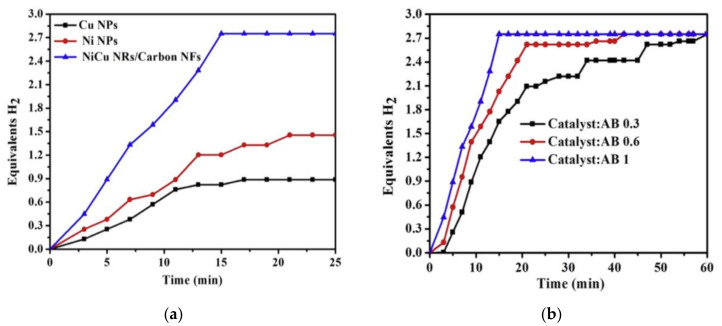
(**a**) Amount of generated H_2_ using Cu NPs, Ni NPs, and electrospun NiCu NRs/C NFs; (**b**) Influence of the ratio of (NiCu NRs/C NFs catalyst to AB) on H_2_ release. Reprinted from reference [54] with permission from Elsevier.

The characterizations of the NFs showed that smooth and continuous polymeric and ceramic NFs were produced before and after calcination, respectively. Amorphous C NFs with a metallic crystalline NiCu NRs alloy was produced. It is very important to note that both Ni and NiCu can catalyze PVP to produce graphite. The catalytic activity of the fabricated NiCu NRs/C NFs was tested and compared with Cu and Ni NPs. As shown in Figure 7a, much higher activity was observed using the fabricated bimetallic catalysts for three main reasons, namely (i) the C NFs’ adsorption capacity, (ii) synergetic effects of NiCu, and (iii) low agglomeration of the NiCu supported on C NFs.

To investigate the influence of catalytic loading on the catalytic performance, the bimetallic catalyst/AB was tested with different ratios (0.3, 0.6, and 1). A higher number of catalysts shortened the H_2_ release time as shown in Figure 7b. The recyclability of the catalyst was studied by testing CuNi NRs/C NFs for six successive cycles. No major change was observed, proving the stability of the catalyst. The activation energy of the catalyst was calculated (28.9 KJ/mol) and found to be lower than many reported noble and non-noble catalysts [57,58,59,60].

### 6.2. Co–TiC NP-Decorated C NFs

Among non-noble metal catalysts, metal carbides have gained incredible interest because of their high melting points, good electronic and magnetic characteristics, high hardness, and corrosion resistance. Titanium carbide (TiC) is one of the promising catalytic carbides due to its chemical and mechanical stability and excellent physical and catalytic properties. Even though TiC has been synthesized and tested for different catalytic reactions, such as the electro-oxidation of AB and NH_3_BH_3_, its synthesis requires extremely high temperatures (up to 1500 °C) to ensure the reaction between the C source and Ti precursor [96].

Aymen et al. have prepared electrospun cobalt–titanium carbide–carbon NFs (Co–TiC–C NFs) at relatively low temperatures using ES, followed by calcination [96]. Co was used for three different reasons. First, to improve the fabrication of C from its polymeric source at moderate temperature in an Ar atmosphere. In fact, the carbonization of a polymer using a catalyst with high catalytic activity such as Co is an effective way to obtain active C. and Second, to lower the calcination temperature and improve the formation of TiC at a moderate temperature by enhancing the reaction between the C source and Ti precursor. Co catalyzed the formation of TiC according to Equation (2). Third, it has a good catalytic activity for H_2_ generation from AB.

6 Ti(OC_3_H_7_)_4_ + C_6_H_9_NO (PVP) → 6 TiC + 6 O_2_ + 2 H_2_ + NH_3_ + 24 C_3_H_6_ + 13 H_2_O (2)

It is worth mentioning that researchers usually use polyacrylonitrile (PAN) as a C NF source [64]. However, other researchers have used PVP as a C source recently because of its solubility in water, cheapness, high surface area, and easy ES ability [65,66].

To prepare Co–TiC NP-decorated C NFs, the ES process, followed by moderate temperature calcination was performed. First, a polymer solution was made by mixing a specific amount of PVP in a mixture of (1:1 wt%) acetic acid (AA) and ethanol at room temperature for an hour. Furthermore, titanium (IV) isopropoxide (TIP) was added dropwise to the previous polymer solution and continuously stirred until the color changed to yellow. A measurement of 10 wt% of CoAc tetrahydrate was further added and continuously mixed with the previous sol–gel for 5 h at 70 °C using an oil bath. ES was then conducted, and the fabricated NFs were vacuum dried for a day at 60 °C. The NFs were finally vacuum dried for 6 h in an Ar atmosphere (heating ramp 2.3 °C/min). To study the effect of the presence of TiC, Co/C NFs were also prepared and compared with Co–TiC NP-decorated C NFs. In addition, to compare Co–TiC NPs with decorated C in particle and fibrous forms, the conventional sol–gel process was utilized to make the particle form using the same recipe.

The authors proposed a mechanism for the formation of Co during calcination as the following:

Co (CH_3_COO)_2_ · 4 H_2_O → (CH_3_COO Co(OH) + 3 H_2_O + CH_3_COOH (3)

(CH_3_COO Co(OH) → 0.5 CoO + 0.5 CoCO_3_ + 0.5 H_2_O (4)

CoCO_3_ → CoO + CO_2_ (5)

CoO + CO → Co + CO_2_ (6)

Co acted as a catalyst to promote the formation of C from PVP (C_6_H_9_NO). Moreover, Co promoted the C and Ti reaction according to the following equation:

6 Ti(OC_3_H_7_)_4_ + C_6_H_9_NO → 6 TiC + 6O_2_ + 2 H_2_ + NH_3_ + 24 C_3_H_6_ + 13 H_2_O (7)

The characterizations of the NFs proved the formation of metallic Co NPs and TiC NPs on C NFs. It was also proved that similar NPs were obtained by calcining the conventional sol–gel.

**Figure 8:**
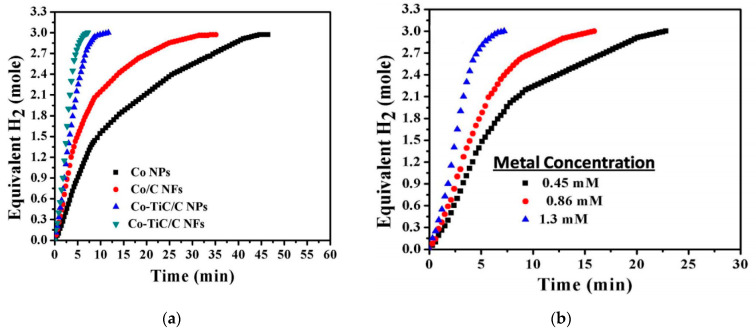
(**a**) Catalytic performance of Co NPs, Co/C NFs, Co-TiC/C NPs, and Co-TiC/C NFs in hydrolysis AB reaction; (**b**) Effect of metal concentration on H_2_ generation. Reprinted from reference [96] with permission from Elsevier.

As shown in Figure 8a, Co–TiC NP-decorated C NFs showed very high activity with a maximum production of three equivalent moles of H_2_ in around 7 min. Co–TiC NP-decorated C NFs produced more H_2_ (0.4167) than Co/C NFs (0.25), Co–TiC/C NPs (0.35), and Co NPs (0.13 mol/min). The authors linked the high performance of the Co–TiC NP-decorated C NFs with the synergetic effects between Co and TiC. Even though the prepared NP catalysts have a greater surface area than the fibrous catalysts, the NFs generated more H_2_ due to the low grain boundaries on them that minimized the agglomeration affinity compared to the NPs.

The authors also studied the effect of the catalyst concentration and temperature on the catalytic performance (Figure 8b). Higher catalytic concentration resulted in a reduction of the H_2_ production time.

**Figure 9:**
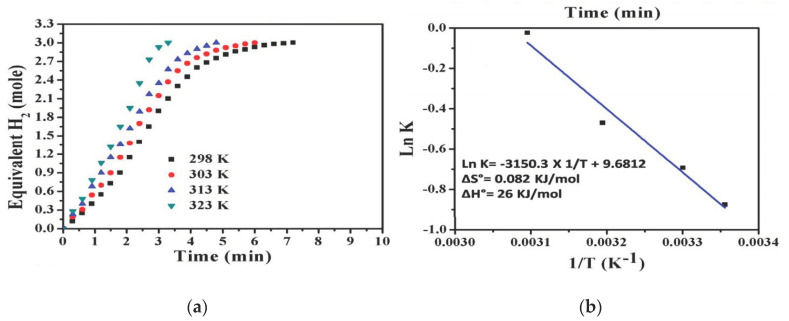
(**a**) Effect of temperature on Co-TiC/C NFs catalyst in hydrolysis AB reaction; (**b**) Plot of ln K vs. (1/T). Reprinted from reference [96] with permission from Elsevier.

The effect of temperature on the catalytic performance was also studied by testing the Co-TiC/C NF catalyst at different temperature (25, 30, 40, and 50 °C). Higher reaction temperatures resulted in a higher reaction rate and lower reaction time as shown in Figure 9a. The activation energy (Ea) was determined from an Arrhenius plot (ln k vs. (1/T), Figure 9b) to be 26.19 kJ/mol, which is higher than most of the reported Ea [9,67,68]. The reaction was found to be a pseudo-first-order reaction concerning the metal concentration.

The recyclability of the catalyst was studied by testing the same catalyst for six successive cycles. No major change in catalytic performance was observed.

To summarize, the high activity of the Co–TiC NP-decorated C NFs was due to four main reasons: (i) Co and TiC synergy, (ii) C NF and TiC chemical stability, (iii) Co presence as an active metal, and (iv) the presence of the C shell layer that protects the NPs from the harsh chemical reaction.

### 6.3. CoCr_7_C_3_-Supported C Nanofibers

Even though titanium carbide (TiC) and tungsten carbide (WC) have been extensively studied as catalysts to replace the precious metallic catalysts, only limited studies have been conducted on chromium carbides (CrCs) [69,70]. CrCs have outstanding electrical conductivity, corrosion resistance, and chemical and mechanical stability. They come in different forms, such as Cr_3_C_2_, Cr_7_C_3_, Cr_23_C_6_, and CrCs.

Ayman et al. have prepared and used a Cr_7_C_3_-supported C NF catalyst to produce H_2_ from AB [9]. Co was added to promote the formation of C from the polymer, catalyze the reaction between C and Cr during the preparation, increase Cr_7_C_3_ durability, and, finally, catalyze AB hydrolysis. Co enhances the formation of metal carbides by (i) facilitating the reaction between the C source and the metal precursor and (ii) lowering the reaction temperature.

To prepare CoCr_7_C_3_-supported C NFs, PVP was first dissolved in a solvent mixture that contained (1:1) AA and ethanol. Chromium (II) acetate dimer monohydrate (CrAc) and CoAc tetrahydrate were dissolved in DI water and mixed with a homogenous PVP solution. The two solutions were stirred for 5 h at 70 °C and further for a day at room temperature. ES was then conducted at 18 kV and at a 15 cm gap distance. The prepared NF mats were dried for a day to remove the solvents. Finally, the mats were calcined for 6 h at 850 °C (heating ramp 2.3 °C/min). For comparison, Co-supported C NFs (prepared) and commercial Co NPs (purchased) were tested and contrasted with the fabricated catalyst.

The characterizations of the prepared catalyst showed the fabrication of smooth and nonbeaded NFs contained Co, Cr_7_C_3,_ and C. The calcination has not influenced the NF morphology due to the polycondensation characteristics of the utilized metal and metal carbides.

**Figure 10:**
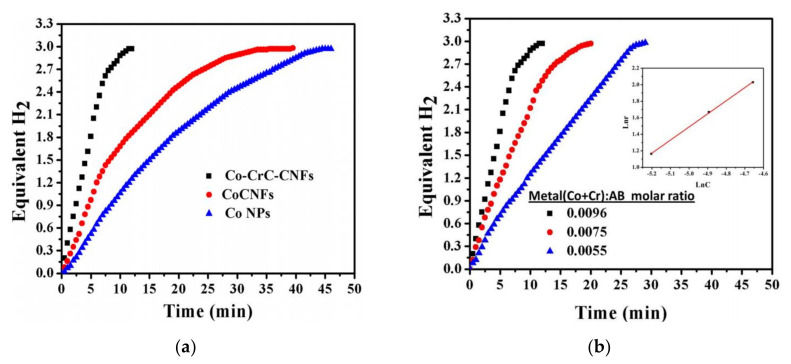
(**a**) Catalytic performance of Co-CrC-NFs, CoC NFs, and Co NPs in hydrolysis AB reaction; (**b**) Effect of metal concentration on H_2_ generation. Reprinted from reference [9] with permission from Elsevier.

As shown in Figure 10a, CoCr_7_C_3_-supported C NFs showed the highest catalytic activity among all tested catalysts. The H_2_ yield was close to the theoretically expected yield. The authors attributed this high activity to (i) Co and Cr_7_C_3_ synergy; (ii) the high adsorption capacity of C, which enhances contacts between AB and the catalysts; and (iii) the high surface area of the fabricated NFs. It is noteworthy that Co-supported C NFs showed higher activity than Co NPs due to the minimum NP agglomeration. The reaction followed a pseudo-first-order concerning the catalyst concentration.

Figure 10b shows the effect of the catalyst concentration on H_2_ generation. Production time was reduced with a higher amount of the catalyst.

The catalyst was tested six times to study its reusability. It showed fair reusability with a gradual decrease in the catalytic activity that reached 61% after the sixth cycle.

### 6.4. NiCr NPs/C NFs

Among first-row transition metals, Ni is an attractive catalyst for AB hydrolysis due to its environmental benignity, good catalytic activity, and ferromagnetic characteristics. However, Ni NP catalysts are easily oxidized in aqueous solutions or air, and they aggregate easily. Hence, their catalytic performance and recycling ability deteriorate quickly [5]. To overcome these issues, a recent study shows the addition of an atomic diffusion barrier (Cr) against Ni atoms to inhibit the agglomeration of Ni. Ni and Cr are neighbors on the periodic table, and they can form a solid solution at room temperature. The NiCr alloy was tested as a catalyst for different reactions and showed promising results [6,97,98]. In a recent work, NiCr NPs were decorated within the surface of C NFs using ES. It is worth mentioning that Ni and its alloys catalyze the formation of C NFs from their polymeric NF source during heat treatment.

To prepare NiCr NP-decorated C NFs, first, a 10 wt% PVP aqueous solution was made at 50 °C [6]. NiAc aqueous solution was then added to the PVP solution. A CrAc aqueous solution was added to the previous solution with different ratios. To obtain a homogenous sol–gel, the solution was stirred for 6 h at 60 °C and subsequently cooled at room temperature. The ES of the sol–gel was then executed at 22 kV and a 20 cm gap distance. The fabricated NFs were vacuum dried overnight at 50 °C. To remove the PVP and convert metal acetates to the desired forms, the polymeric sample was sintered for 5 h at 800 °C (3 °C/min) under an Ar atmosphere.

The characterizations of the NFs showed the fabrication of smooth and nonbeaded NFs. The uniform structure was due to the polycondensation between the NiAc and CrAc with PVP. It was confirmed that the heat-treated sample was composed of Cr_2_Ni_3_ alloy NPs decorated on C NFs.

**Figure 11:**
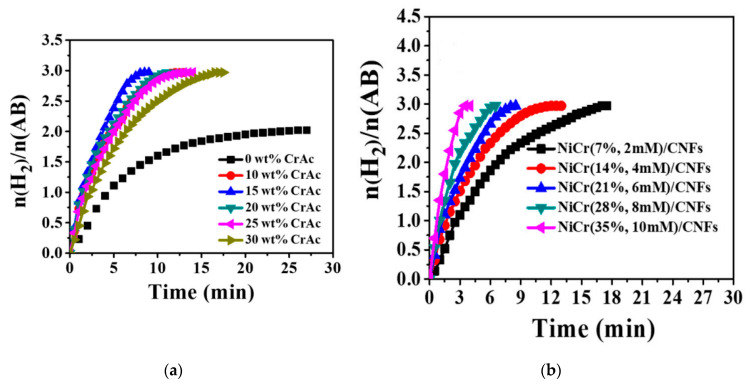
(**a**) Catalytic performance of Ni with different amount of Cr supported on C NFs in hydrolysis AB reaction; (**b**) Effect of metal concentration on H_2_ generation. Reprinted from reference [6] with permission from MDPI.

The authors performed different tests to study the catalyst performance. Due to the synergetic effects between Ni and Cr, the bimetallic NiCr/C NFs showed higher activity than the pure Ni/C NFs. For example, the bimetallic catalysts produced two moles of H_2_ per each mole of AB in a time shorter than that of the mono catalyst by 27 min. A higher amount of Cr leads to a higher activity of NiCr. As shown in Figure 11a, the highest catalytic activity was achieved with the bimetallic Cr/Ni composed of 15 wt% Cr and 85% Ni supported on C NFs. The excellent activity of the proposed catalyst was attributed to the following reasons: (i) Co increases the surface area and functions as an atomic barrier to prevent Ni agglomeration; (ii) the interaction of Cr and Ni leads to the formation of an alloy with a modified electron structure; (iii) the existence of a large number of active sites due to the high surface area of C NFs, the high adsorption capacity of C NFs, and good interaction between the well-dispersed NiCr and CNFs; and (iv) the ability of C NFs to facilitate the interaction between AB and NiCr due to their nonporous structure, which helps to adsorb AB efficiently. It is obvious from Figure 11b that a higher amount of the catalysts produces higher and faster H_2_.

The effect of temperature on the performance of 15 wt% NiCr NPs/C NFs was also studied, and the time duration for H_2_ generation was reduced from 12.5 min to 3.5 min when the temperature was increased from 25 °C to 50 °C. The catalyst was tested five times to study its reusability, and a gradual decline in its performance was observed (around 65% at the fifth run). The authors believe that the decline is due to the following reasons: (i) the partial clustering of NiCr, (ii) the increase in the solution viscosity, and (iii) the deactivation of the active sites due to the accumulation of B products.

### 6.5. TiO_2_ NFs as a Catalytic Support for AB Hydrolysis Reaction

TiO_2_ NFs have important applications in different fields, including catalysis. They are used as a catalyst and a photocatalyst. Most of the TiO_2_ NF-supported catalysts were utilized as photocatalysts in the AB reaction; hence, photocatalysis will be explained in more detail before the discussion of the electrospun photocatalysts.

### 6.6. Co–B Nanoflakes (NFlks)/TiO_2_ NFs

As shown previously, it is accepted that Co is one of the promising catalysts that may replace the expensive precious metals for AB hydrolysis. The development of an amorphous Co–B catalyst showed higher H_2_ release than the crystalline Co–B and Co powder. Co–B possesses unique properties, such as a high concentration of coordinatively unsaturated sites, isotropic structure, and chemical stability. Co–B nanocatalysts are more active than regular Co–B catalysts due to their higher surface area and porosity. Moreover, nanoalloy catalysts have short-range order and long-range disorder. Despite all these good catalytic properties, the regular and nanocatalyst Co–B suffer from aggregation in AB hydrolysis due to the magnetic properties of the catalyst and the nature of the hydrolysis process. To overcome the agglomeration issue, Yousef et al. have synthesized Co–B nanoflakes (NFlks) supported on TiO_2_ NFs and tested their performance in the absence of light [99].

To prepare the catalyst, first, TiO_2_ NFs were prepared by mixing PVP with (1:1 wt%) AA and ethanol for 1 h at room temperature. Furthermore, titanium isopropoxide (TTIP) was added to the PVP solution and stirred until the solution color turned yellow. Subsequently, ES was conducted to produce a fibrous mat. The NF mat was then dried to remove the solvent and treated with heat at 700 °C for 1 h.

To prepare Co–B NFlks/TiO_2_, the fabricated TiO_2_ NFs were mixed with DI water and CoAc. The mixture was sonicated for 30 min and stirred at 50 °C for 1 h to ensure the CoAc dissolved completely. Furthermore, aqueous NH_3_BH_3_ was added dropwise at low temperatures to prevent a vigorous reaction. To obtain better Co–B attachments on the surface of TiO_2_ NFs, the participates were sonicated for 10 min after the NH_3_BH_3_ reaction. The colloidal solution was washed with DI water three times to remove impurities. The residue was vacuum dried for a day at 80 °C. For comparison, a Co–B powder was prepared under the same conditions.

**Figure 12:**
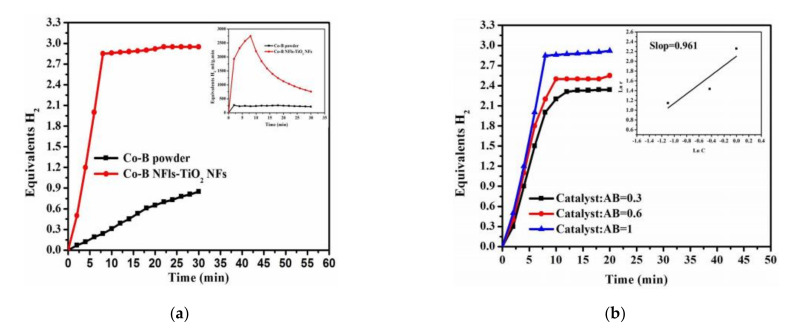
(**a**) Catalytic performance of Co-B NPs and Co-B NFs-TiO_2_ NFs in hydrolysis AB reaction. (**b**) Effect of metal concentration on H_2_ generation. Reprinted from reference [99] with permission from Elsevier.

The characterizations of the fabricated catalyst showed the growth of amorphous Co–B NFlks on the surface of crystalline TiO_2_NFs. The catalytic activity was highly affected by the Co–B structure. Co–B NFlks/TiO_2_ showed much higher activity than the Co–B powder for AB hydrolysis, as shown in Figure 12a, and a higher amount of the NF catalysts leads to high H_2_ in a shorter time (Figure 12b). H_2_ was immediately generated when Co–B NFlks/TiO_2_ were added to the AB aqueous solution. The reaction followed pseudo-zero-order kinetics for AB concentration and pseudo-first-order for the catalyst concentration.

The recyclability of the nanocatalysts was studied by testing the same catalyst for six successive cycles without regeneration. A small H_2_ release decrease was observed due to the decrease in the surface activation of the catalyst.

## 7. Photocatalysis

Over the past 4 decades, a considerable amount of effort has been made and interest has been shown for heterogeneous photocatalysis [100,101], especially for their environmental and energy applications. Photocatalytic chemical reactions are reactions initiated by the absorption of light (photons), and theses reactions include many different chain reactions.

Photocatalysts are catalysts that increase the rate of reaction in the presence of light. Photocatalysis is mainly based on materials that can create electrons and holes when subjected to light. The generated electrons and holes form radicals in different pathways. Heterogeneous AB hydrolysis photocatalytic reactions normally include the following steps:

i. Irradiation of the semiconductor catalyst;
ii. Electronic transition of electrons from the valance band to the conduction band of the semiconductor;
iii. Creation of holes in the valance band because of the electronic transition of electrons;
iv. Generation of radicals from electrons and holes;
v. Reaction of AB with radicals to generate H_2_.

**Figure 13:**
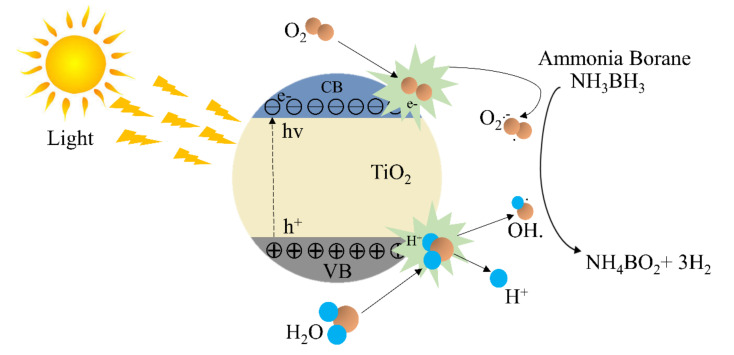
Photocatalytic steps for AB hydrolysis.

Figure 13 shows the photocatalytic steps for AB hydrolysis. To design effective photocatalysts, certain characteristics of the photocatalytic materials must be considered. These characteristics include bandgap, carrier transport, crystallinity, surface area, and chemical stability.

Band gap is the gap between the valance and conduction bands. Materials with very high bandgaps, such as insulators, are not recommended for use as photocatalytic materials. Light with very high energy is needed to excite materials with very high bandgaps. Thus, materials with very high bandgaps cannot function well with free solar energy. Solar radiation has a distribution of frequencies, and most of the frequencies lie in the visible and ultraviolet (UV) regions. Moreover, materials without bandgaps such as metals are not good photocatalytic materials since their conduction and valance bands overlap. This overlapping allows electrons and holes to recombine easily. These kinds of materials only allow oxidation or reduction to occur, and this is not practical in photocatalysis since both reactions must occur simultaneously. Hence, ceramic semiconductor materials are the best choice for photocatalytic applications due to their reasonable bandgap. Carrier transport is another important factor since there is some transportation of electrons and holes.

The crystallinity of a material is a measure of the degree of structural order. It affects the bandgap and carrier transport.

The surface area is a very crucial aspect in all kinds of catalysts. Higher surface area leads to higher active sites, which increase the rate of the chemical reaction. Chemical stability is a measure of the life cycle of the photocatalyst. Photocatalysts must maintain their photocatalytic activity for a very long time to be economically beneficial. Generally, in addition to the characteristics of traditionally known good catalysts, an efficient photocatalyst must has the following two important criteria: (i) a photocatalyst must form electrons and holes efficiently when exposed to light and (ii) the generated electrons and holes must be kept separated from each other to prevent recombination. Charge separation is a very essential property to be considered when designing new photocatalytic materials.

### 7.1. Titanium Dioxide Photocatalysts

It is well known that titanium dioxide (TiO_2_) is the most famous photocatalytic material. TiO_2_ was first used as a photocatalyst in 1972 by Fujishima and Honda for splitting water [102]. Since that discovery, TiO_2_ has continued to be researched extensively as an industrial catalytic and photocatalytic material in different applications, such as water treatment, biomedical equipment, the fine chemical industry, and energy.

TiO_2_ is an active, cheap, chemically inert, stable, and environmentally friendly photocatalyst with strong oxidizing powers [7,103]. However, the main issue of TiO_2_ is its relatively large bandgap that prevents its effective photocatalytic activity under visible light. The photocatalytic activity of TiO_2_ is high when the catalyst is subjected to light close to the UV region, which only allows the harvesting of about 5% of the free available incident solar radiation.

Based on crystallinity, TiO_2_ has an amorphous form and three different crystalline forms, namely anatase, brookite, and rutile. The crystalline structure of anatase and rutile corresponds to the tetragonal system, while brookite has an orthorhombic crystalline structure. Even though rutile, as a bulk material, is the stable phase, anatase is desirable. Brookite and anatase have a metastable phase and readily transform to rutile when heated [104].

**Figure 14:**
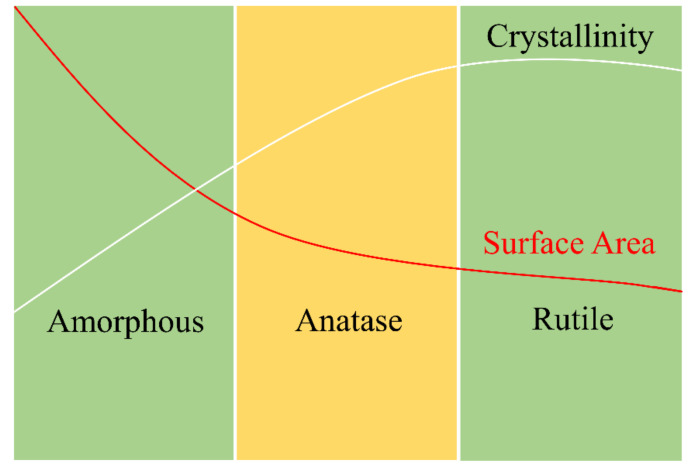
Effect of calcination temperature on TiO_2_ crystallinity and surface area.

Generally, TiO_2_ is prepared from an alkoxide precursor, TTIP. The alkoxide is hydrolyzed with water to obtain TiO_2_·nH_2_O hydrates at low temperatures. This hydrate is amorphous and has a high surface area. The calcination of TiO_2_·nH_2_O at medium and high temperatures forms anatase TiO_2_ and rutile TiO_2_, respectively. A higher temperature also results in higher crystallinity and lower surface area. Figure 14 shows the effect of calcination temperature on TiO_2_ crystallinity and surface area. A higher surface area implies higher active sites, which result in higher reaction activity. A high crystallinity provides higher mobility of carriers with lower recombination probability. The bandgaps of anatase and rutile TiO_2_ are 3.2 and 3.0 eV, respectively. It is obvious that anatase is the best TiO_2_ form for photocatalysis since it provides a balance between crystallinity and surface area, leading to high photocatalytic activities.

### 7.2. Modification of TiO_2_ for Higher Photocatalytic Activity under Visible Light

TiO_2_ is an efficient photocatalyst only under UV light. This is not practical from an economic point of view, as the catalysts should be active under free visible light. Hence, TiO_2_ should be modified to be active under visible light. Different methods have been applied to modify TiO_2_ for higher photocatalytic activity under visible light. Table 3 summarizes some of the TiO_2_ modification procedures [103,105].

Generally, anatase TiO_2_ in one-dimensional (1-D) nanostructures (NRs, nanowires, nanobelts, nanotubes, or NFs) has wide applications as a photocatalyst. Among all 1-D nanostructural TiO_2_, NFs have a large axial ratio (length to diameter ratio) and a low number of grain boundaries. These NF properties provide a fast charge transfer for better photocatalytic performance. However, even anatase TiO_2_ NFs have insufficient catalytic activity under sunlight and must be modified. The modification of electrospun TiO_2_ NFs with metallic and nonmetallic materials has been accomplished by many researchers, as shown in the following section [106,107].

## 8. TiO_2_ NFs as a Catalytic Support for AB Hydrolysis Reaction

Different electrospun ceramic catalysts have been prepared and tested in the presence of light as photocatalysts for AB hydrolysis. Table 4 shows the important data of each electrospun photocatalyst, and the details are explained in the following sections.

### 8.1. Ni-Doped TiO_2_ NFs

Nirmala et al. published the first electrospun TiO_2_ NF photocatalyst for AB hydrolysis in 2012 [103]. To prepare the modified TiO_2_ catalyst, first, PVP was mixed with ethanol for 3 h at room temperature. Furthermore, a TiO_2_ solution was made by dissolving TIP in a mixture of AA and ethanol (2:3:5 vol). The sol–gel solution was made by mixing PVP and TiO_2_ solutions for 30 min. Subsequently, NiAc tetrahydrate was dissolved in ethanol, added to the sol–gel, and mixed for 30 min to ensure perfect dispersion. The ES of the sol–gel was executed under 15 kV and at 15 cm, before and after adding NiAc. The fabricated fibrous mats were vacuum dried for a day at 50 °C, followed by calcination in an Ar atmosphere for 3 h at 550 °C (5 °C/min).

Smooth NFs with uniform diameters were observed with 150–200 nm and 100–150 nm diameter ranges before and after calcination, respectively. TiO_2_ was observed in both rutile and anatase phases, with rutile as the dominant phase. Ni NPs appeared after calcination, proving the successful fabrication of well-defined and separated spherical Ni NPs/TiO_2_ NFs without any other impurities.

**Figure 15:**
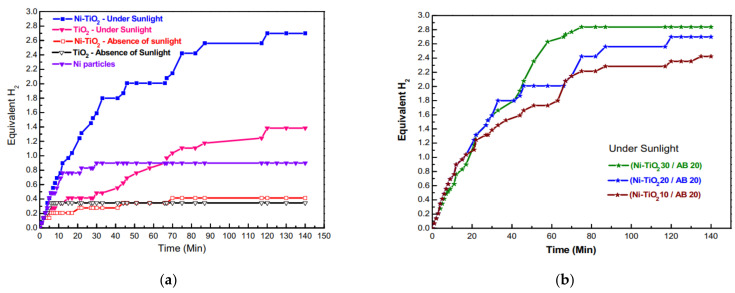
(**a**) Catalytic performance of Ni-TiO_2_ NFs in the absence and presence of light for AB hydrolysis reaction; (**b**) Effect of metal concentration on H_2_ generation. Reprinted from reference [103] with permission from Elsevier.

The catalytic performance was studied for TiO_2_ NFs and Ni NPs/TiO_2_ NFs under sunlight and indoor daylight. The performances of electrospun catalysts were also compared with nonsupported Ni NPs. As shown in Figure 15a, the catalytic performance was in the following order: Ni NPs/TiO_2_ NFs (sunlight) > TiO_2_ NFs (sunlight) > Ni NPs (sunlight) > Ni NPs/TiO_2_ NFs (daylight) > TiO_2_ NFs (daylight). It is very important to note that the amount of nonsupported Ni NPs was almost 20 times the amount of Ni NPs in the supported catalyst (Ni-TiO_2_). H_2_ generation was much faster and higher in sunlight, proving the photocatalytic activity of the fabricated catalysts. A very limited amount of H_2_ was produced using either the doped or pristine TiO_2_ NFs under daylight due to the minimum photoexcitation reaction.

As shown in Figure 15b, increasing the amount of Ni/TiO_2_ (10–20–30 mg) under sunlight led to a slight increase in the H_2_ generation, indicating the high efficiency of the catalyst. The highest H_2_ production was around 2.8 equivalent moles of H_2_ when using 30 mg of Ni/TiO_2_ and 20 mg of AB.

### 8.2. Ni(0)-Doped TiO_2_/C NFs

Ni NPs were used to enhance the photocatalytic activity of TiO_2_ NFs in [80,81]. However, the authors have also incorporated C NFs in the Ni/TiO_2_ for better performance. The preparation steps were different from those in the previous study, especially the calcination atmosphere [15].

Ni(0)-doped TiO_2_/C NFs were prepared by dissolving TIIP in a mixture of AA and ethanol. After stirring the previous solution for 15 min, ethanol and PVP were added. Furthermore, the mixture was stirred continuously until a transparent yellow sol–gel was achieved. NiAc was then added to the sol–gel and the solution was stirred well. The ES of the sol–gel was executed at 20 kV and 15 cm. The fabricated fibrous mat was vacuum dried at 60 °C for a day. Calcination was performed in a 1:1 Ar/H_2_ atmosphere for 5 h at 700 °C (2.3 °C/min heating rate). The Ar/H_2_ was used as a calcination atmosphere to maintain the NF morphology and minimize the rate of polymer decomposition. Pristine TiO_2_/C NFs were prepared using the same procedure but without adding NiAc.

The characterization of the electrospun samples showed the fabrication of continuous bead-free and smooth NFs before and after calcination. After calcination, the cubic crystal structure of Ni(0) NPs was formed on tetragonal anatase and rutile TiO_2_ and C NFs. C and TiO_2_ formed a core-shell structure with graphite at the shell, which helped in protecting the catalyst and adsorbing AB. It is noteworthy that Ni(0) catalyzed the graphitization of PVP.

**Figure 16:**
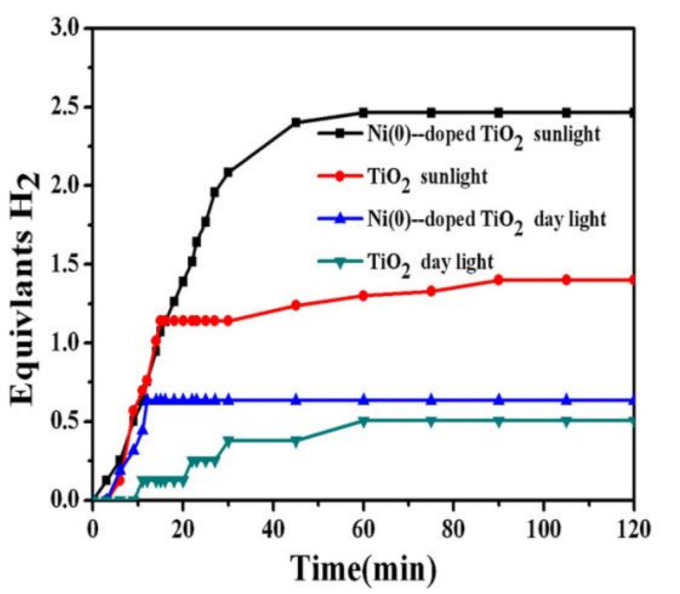
Catalytic performance of TiO_2_ NFs with and without Ni(0) in sunlight and daylight for AB hydrolysis reaction. Reprinted from reference [15] with permission from Elsevier.

Four major tests were performed to study the catalytic performance, namely those based on Ni(0)-doped TiO_2_ in sunlight and daylight and pristine TiO_2_ in sunlight and daylight. As shown in Figure 16, the activities of the tested catalysts were in the following order: Ni(0)-doped TiO_2_ (sunlight) > TiO_2_ (sunlight) > Ni(0)-doped TiO_2_ (daylight) > TiO_2_ (daylight). The highest activity was achieved using Ni(0)-doped TiO_2_ in sunlight with 2.5 equivalent moles of H_2_ in an hour. The authors argued that Ni(0) NPs have a dual effect. First, Ni reduces the recombination rate of electrons and holes, which increases the intensity of the ions in the solution. Second, it catalyzes the AB hydrolysis reaction.

### 8.3. CuO/TiO_2_ NFs

Cu-based NP catalysts have shown good activity toward AB hydrolysis. CuO is the most common type of copper and can be used to hydrolyze AB. However, the agglomeration of CuO NPs hinders its widespread usage. Yousef et al. have overcome this dilemma by embedding CuO NPs inside TiO_2_ NFs [16]. To prepare the catalyst, first, TIIP was dissolved in a mixture of AA and ethanol and stirred for 15 min. Furthermore, PVP and ethanol were stirred into the previous mixture until a transparent yellow sol–gel was obtained. Cu NPs were then added to the sol–gel and mixed to ensure that they were well dispersed. The ES of the prepared sol–gel was performed at 20 kV and a 15 cm gap distance. The fabricated electrospun samples were vacuum dried at 60 °C for a day. The calcination of the dried samples was executed under an air atmosphere at 700 °C for 1 h, with a heating ramp of 5 °C/min. Pristine TiO_2_ was prepared using the same steps but without adding Cu NPs. Cu NPs were also calcined under the same calcination conditions and used for comparison.

Good NF morphology was observed before and after calcination. Tetragonal rutile and anatase TiO_2_ NFs were observed after the calcination of the Cu-free electrospun mat. Additionally, CuO NPs were achieved after the calcination of Cu NPs at 700 °C. The same phases were obtained after the heat treatment of the Cu–PVP–TIIP sample, leading to the formation of high crystalline CuO NPs/TiO_2_ NFs. Based on the crystal lattice parameters of CuP and TiO_2_, they cannot combine in a single structure.

The catalysts were initially tested in daylight, and the activity of the catalysts was in the following order: CuO NPs/TiO_2_ NFs > TiO_2_ NFs > CuO NPs. Furthermore, 2.7 moles of an H_2_ equivalent was generated in 15 min. The effects of temperature on the catalytic performance were studied, and the calculated activation energies were 44.8 (CuO NPs/TiO_2_ NFs), 53.6 (TiO_2_ NFs), and 75.2 kJ/mol (CuO NPs). CuO NPs/TiO_2_ NFs showed great stability since their performance was fixed for three successive cycles. Hence, the agglomeration of CuO NPs was minimized when the NPs were supported on TiO_2_ NFs.

Before testing the photocatalytic activity of the prepared catalyst, a photoluminescence study was performed. Photoluminescence is used to study the catalyst’s ability to trap, migrate, transfer, and separate charge carriers and to study the lifetime in the catalyst. It was found that the photoluminescence intensities decreased after the addition of CuO to TiO_2_. A lower intensity implies a lower recombination of electrons and holes.

**Figure 17:**
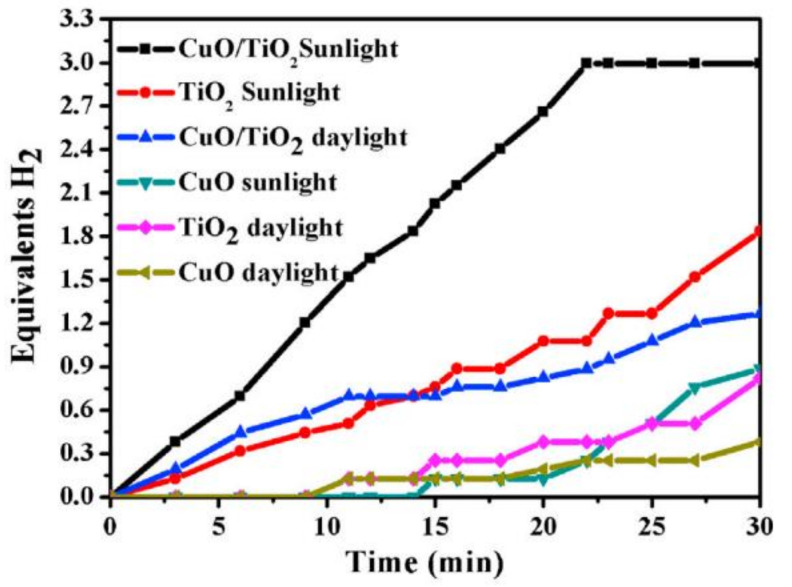
Catalytic performance of TiO_2_ NFs with and without the incorporation of CuO in daylight and sunlight for AB hydrolysis reaction. Reprinted from reference [16] with permission from Elsevier.

The photocatalytic study was performed by testing the three catalysts (CuO, TiO_2_, and CuO/TiO_2_) under sunlight and daylight. Since TiO_2_ is highly affected by the presence of light, both CuO/TiO_2_ and pristine TiO_2_ showed much higher activity in sunlight compared to daylight as shown in Figure 17. Naked CuO NPs also performed a little better in the presence of light. The highest photoactivity was achieved with CuO NPs/TiO_2_ NFs, generating the maximum theoretical H_2_ in 20 min. Hence, the photocatalytic activity of TiO_2_ for AB hydrolysis was greatly enhanced by the addition of CuO.

### 8.4. Cu_(0)_ NPs/TiO_2_ NFs

Ayman et al. have studied Cu_(0)_ NPs on the surface of TiO_2_ NFs and tested their performance for the generation of H_2_ from AB. Zero-valent copper (Cu_(0)_) was used as a dopant chemical to overcome the issue of the large bandgap of TiO_2_ [14]. Cu_(0)_ has many salient characteristics such as relative availability, minor toxicity, and cheapness compared to noble metals. Cu_(0)_/TiO_2_ was prepared through a combination of ES and a hydrothermal technique. First, TiO_2_ NFs were prepared by dissolving TIIP in a solvent mixture of AA and ethanol (1:1 wt.%). The mixture was stirred for 15 min and added to another mixture containing PVP and ethanol. The stirring of the sol–gel mixture was performed until a homogenous yellow mixture was obtained. The yellow solution was then subjected to ES at 20 kV with a 15 cm gap. The electrospun NFs were vacuum dried at 60 °C for a day to remove the residual solvents. The calcination of the dried sample was executed at 700 °C for 1 h to remove the polymer and obtain the ceramic TiO_2_.

The hydrothermal process was used to introduce metallic Cu to the fabricated crystalline TiO_2_ NFs. Copper nitrate was dissolved in a mixture of ethanol and water (95:5 vol%). The previous mixture was subsequently mixed with another mixture made from formic acid, ammonia solution, and the fabricated electrospun TiO_2_ NFs. The Cu mixture and the NFs mixture were mixed for 1 h to ensure high dispersion. The obtained blue solution was transferred into a tightly sealed stainless-steel autoclave. The autoclave was operated at 170 °C for 3 h and cooled to room temperature. A white powder suspended in a pink solution was obtained. The powder was filtered using a filter paper, placed in a glass bottle filled with distilled water, and sonicated for 2 min. The sonication step was performed to remove the suspended particles. After the sonication, the powder was washed with water and ethanol several times and dried at 60 °C for 24 h. The same hydrothermal process was repeated to prepare nonsupported Cu particles for use in catalytic comparison.

The characterizations of the final catalyst proved the formation of Cu_(0)_ NPs on the surface of nanoporous and rough TiO_2_ NFs. It was also observed that the inner surface of TiO_2_ NFs was also nanoporous, and the authors attributed this to the strong hydrothermal reaction. TiO_2_ NFs existed in both the anatase and rutile phases.

The mechanism of the reduction of the Cu precursor (Cu nitrate) to Cu_(0)_ on the surface of TiO_2_ NFs during the hydrothermal process was suggested as follows: (i) the formic acid reduced Cu nitrate to Cu ions; (ii) a stronger electrostatic attraction between TiO_2_ NFs and Cu^2+^ ions occurred due to the increase in the negative charge of TiO_2_ NFs, while the negative charge increase took place due to the increase in the potential of hydrogen (pH) of the solution after the hydrothermal process; and (iii) Cu^2+^ ions were oxidized to metallic Cu on the surface of the NFs according to the following equations:

4 OH^−^ → 2 H_2_O + O_2_ + 4 e^−^ (8)

2 Cu_2_+ + 4e^−^ + 2 Ti-O^−^ → 2 Cu_(0)_TiO_2_ (9)

**Figure 18:**
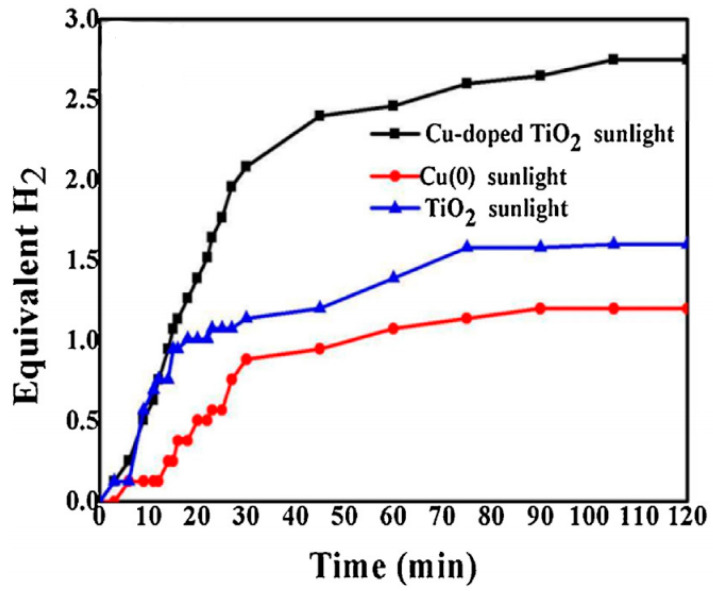
Catalytic performance of TiO_2_ NFs with and without the incorporation of Cu_(0)_ in sunlight for AB hydrolysis reaction. Reprinted from reference [14] with permission from Elsevier.

Cu_(0)_/TiO_2_ NFs exhibited higher photocatalytic activity than pure TiO_2_ NFs and Cu_(0)_ NPs toward H_2_ generation from AB as shown in Figure 18. H_2_ yields were 23% (Cu_(0)_ NPs), 53% (TiO_2_ NFs), and 90% (Cu_(0)_ NPs/TiO_2_ NFs). The authors linked the high activity of Cu_(0)_ NPs/TiO_2_ NFs to two main reasons: (i) Cu_(0)_ NPs, which decreased the electrons–holes recombination rate and worked as a sink for the generated photoelectrons from TiO_2_ NFs, attacking the dihydrogen bonds between AB molecules and breaking them and (ii) the synergistic effects of Cu_(0)_ and TiO_2_ NFs. The catalyst exhibited very good catalytic stability since no obvious performance decrease was observed when the same catalyst was used thrice.

### 8.5. Cu-Doped TiO_2_/C NFs

Wu et al. have studied the influence of Cu and C doping on TiO_2_ NFs separately. The photocatalytic activity of the Cu and C-doped TiO_2_ was promising [114]. However, since the simultaneous doping of Cu and C on TiO_2_ NFs is challenging, limited work has been published on Cu- and C-doped TiO_2_ NFs.

**Figure 19:**
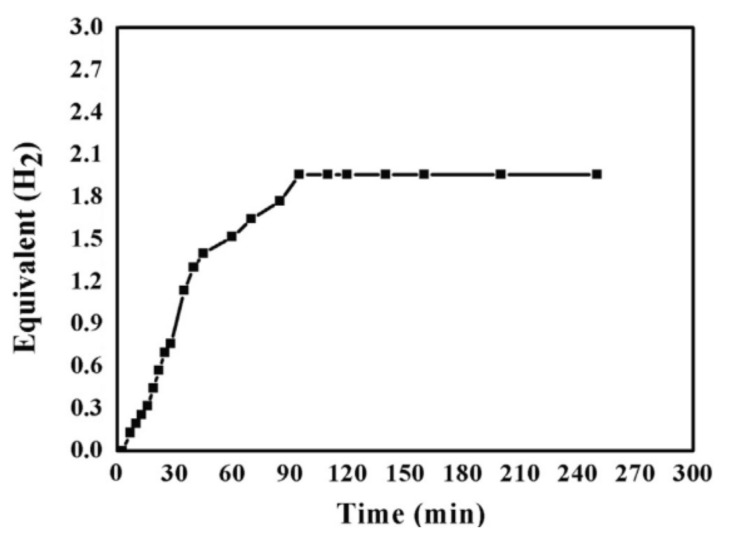
Catalytic performance of Cu-doped TiO_2_ carbon nanofibers for AB hydrolysis reaction under sunlight. Reprinted from reference [111] with permission from Elsevier.

After their success in doping TiO_2_ NFs with Cu, Yousef et al. conducted another study to support the use of Cu and C on TiO_2_ NFs in one pot for the first time [111]. Simultaneous Cu and C doping on TiO_2_ NFs was achieved using ES. First, TIIP was mixed with PVP and a mixture of (1:1 wt%) AA and ethanol. The sol–gel was mixed until it became transparent. Furthermore, Cu(II) acetate tetrahydrate was added to the previous solution and stirred for an hour at 50 °C to ensure it was mixed well. After that, ES was executed at 18 kV and a 15 cm gap distance. Electrospun NFs were dried at 60 °C for a day to remove the solvents. The calcination was finally performed in an Ar atmosphere for 2 h at 750 °C with a 2.3 °C/min heating rate, followed by cooling the mixture to room temperature. The characterizations of the NFs proved the fabrication of bead-free, continuous, and smooth NFs. The NFs contained cubic crystalline Cu^0^, tetragonal anatase TiO_2,_ and amorphous graphite. Graphite existed as a thin layer covering the NFs. The photocatalytic activity of the prepared one-pot Cu NP-doped TiO_2_ C NFs was tested under sunlight. Cu NP-doped TiO_2_ C NFs showed excellent photocatalytic activity since a fast H_2_ release rate was obtained under sunlight as shown in Figure 19.

### 8.6. Cu^0^/S-Doped TiO_2_ NPs/C NFs

Yousef et al. have improved their Cu–TiO_2_ C NFs catalyst by the addition of sulfide (S) to obtain Cu^0^/S–TiO_2_ NPs/C NFs [8]. To fabricate the catalytic NFs, first, PVP was mixed with AA and ethanol. Further, TIIP was added to the previous solution and continuous mixing was performed until a yellow solution was achieved. CuAc and ammonium sulfide were then added (dropwise) to the PVP–TIIP solution and stirred for 12 h. The ES of the well-mixed sol–gel was performed at 20 kV. The fabricated NFs were vacuum dried for 12 h. Finally, the calcination of the dried NFs was executed for 3 h at 800 °C in an Ar atmosphere. For comparison, TiO_2_–C NFs and Cu–TiO_2_/C NFs were prepared following the same steps, except for the calcination, which was performed in an air atmosphere for 1 h at 600 °C.

**Figure 20:**
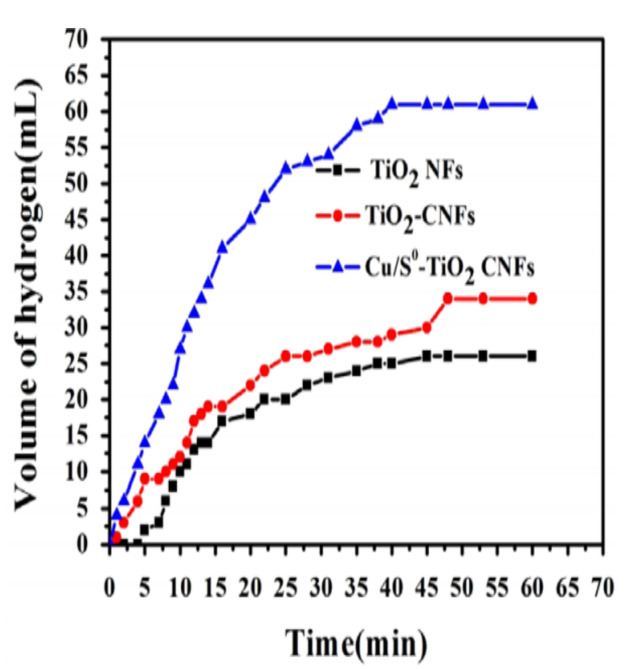
Photocatalytic performance of TiO_2_ NFs with and without Cu/S for AB hydrolysis reaction. Reprinted from reference [8] with permission from Elsevier.

Organic and inorganic NFs with good morphology were observed before and after heat treatment, respectively. The high-temperature calcination of the synthesized electrospun NFs led to (i) the decomposition of PVP and the formation of TiO_2_ and (ii) the thermal decomposition of CuS to Cu^0^ and S. A Cu^0^/S-doped TiO_2_ NP-decorated NF catalyst was formed, and the C was amorphous graphite-like. The performance of the prepared Cu^0^/S–TiO_2_ NPs/C NF photocatalyst was compared with other nanofibrous catalysts (Cu–TiO_2_/C NFs, TiO_2_–C NFs, and TiO_2_ NFs). As shown in Figure 20, Cu^0^/S–TiO_2_ C NFs showed better photocatalytic activities than the other tested catalysts (the H_2_ yield was 91%, 60%, 49%, and 38% using Cu^0^/S–TiO_2_ C NFs, Cu–TiO_2_ C NFs, TiO_2_–C NFs, and TiO_2_ NF catalysts, respectively). Thus, the synergistic effect of Cu and S was confirmed on TiO_2_ NPs for AB hydrolysis because of the enhanced activity of the Cu^0^/S–TiO_2_ C NFs over other tested catalysts. Moreover, the authors believe that the existence of C helped to increase the H_2_ evolution due to the high adsorption capacity and fast electron transfer.

The activity of the catalyst decreased when the same catalyst was used for a second and third cycle (the efficiency dropped from 100% to 80%, and then to 60%, respectively). The authors proposed the following mechanism:

i. The generation of electrons during the photocatalytic process.
ii. The transfer of electrons from S-doped TiO_2_ NPs to the vacant Cu d-orbital, leaving holes in the S-doped TiO_2_ NPs.
iii. The adoption of AB by C NFs and the transfer of electrons in Cu to H_3_N^2+^, according to Equation (2).
iv. Positive charges in the S-doped TiO_2_ NPs attacked H_3_B_2_^-^ forming three moles of H_2_.

### 8.7. CdS–TiO_2_-Doped C NFs

Photocatalysts made of photolytic semiconductors such as TiO_2_ and CdS supported on C substrates are of great interest. C NFs are a form of C, which demonstrate the efficient capture and transport of photogenerated electrons [110]. Cadmium sulfide (CdS) is another important photolytic material. It is a sulfide semiconductor that is used as a photocatalyst due to its (i) ideal bandgap (2.4 eV), which makes CdS photocatalytically active in visible light regions, and (ii) appropriate conduction band (CB) edge compared to the reduction potential of H_2_. However, pure CdS suffers from photo corrosion and has a high recombination rate and low surface area.

As the CdS CB is higher than that of TiO_2_, it is believed that the electrons from CdS that are excited by visible light are quickly transferred to TiO_2_. This electron transportation results in a more condense CB of TiO_2_.

Pant et al. have fabricated an electrospun CNF-decorated CdS/TiO_2_ heteroarchitecture for visible light photocatalytic AB hydrolysis [112]. Four different electrospun photocatalysts (TiO_2_, TiO_2_–C NFs, CdS–C NFs, and CdS–TiO_2_–C NFs) were prepared and compared to each other. The precursors of TiO_2_, C NFs, Cd, and S were titanium tetraisopropoxide (TTIP), PVP, cadmium acetate (CdAce), and ammonium sulfide (AS), respectively.

To fabricate these photocatalysts, first, TTIP and AA were mixed for 10 min. Furthermore, ethanol and PVP were added to the previous solution, followed by the addition of CdAc and AS (dropwise). Each precursor was added depending on the desired final photocatalyst. The final mixtures (TTIP + PVP for TiO_2_ and TiO_2_–C NFs; PVP + CdAc + AS for CdS–C NFs; and TTIP + PVP+ CdAc + AS for CdS–TiO_2_-C NFs) were stirred for 12 h at room temperature to obtain homogenous ES solutions. All solutions were then subjected to ES at 20 kV at a 15 cm gap distance to fabricate the polymeric NF mats. Finally, the calcination of the resultant electrospun mats was executed in an Ar atmosphere at 600 °C for TiO_2_ and 850 °C for other catalysts.

The characterizations of the fabricated fibers before calcination confirmed the formation of bead-free, continuous, and smooth NFs. It is known that the rate of polymer decomposition of the electrospun NFs is reduced when they are treated with heat in an Ar/H_2_ isolated system. However, in this study, the morphology of the NFs was maintained. Cds–TiO_2_/C NFs with continuous structures and small-fiber diameters were formed after calcination at 850 °C. The authors believe that the formation of crystalline TiO_2_ and CdS NPs on the surface of the amorphous C NFs occurred during the calcination process. It was concluded that PVP was converted into the well-known defects (D) and graphite (G) bands of graphite after calcination, and the final CdS–TiO_2_–C NFs had around 60% carbon.

**Figure 21:**
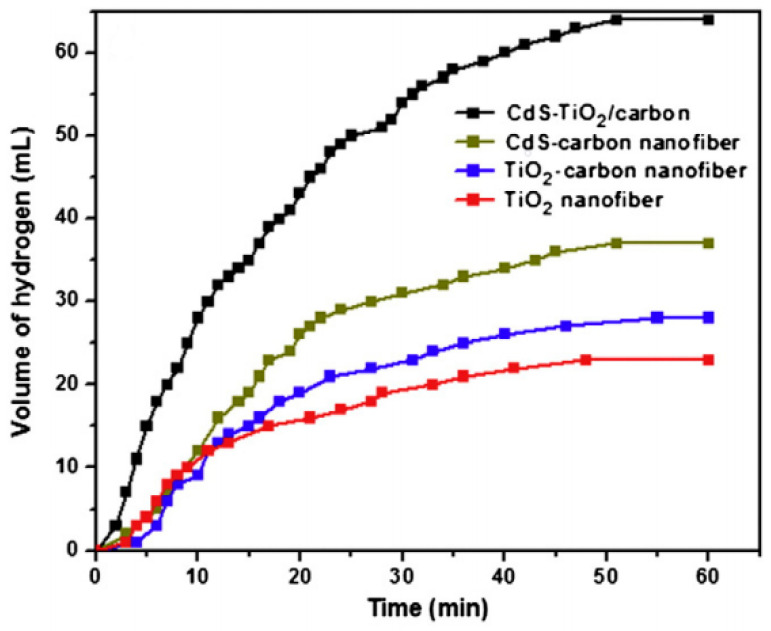
Photocatalytic performance of TiO_2_ NFs with and without the incorporation of CdS and C NFs for AB hydrolysis reaction. Reprinted from reference [112] with permission from Elsevier.

The prepared catalysts were tested for the photohydrolysis of AB under visible light irradiation. The H_2_ yield was measured and found to be around 34% (TiO_2_ NFs), 40% (TiO_2_–C NFs), 55% (CdS/C NFs), and 95% (CdS–TiO_2_/C NFs). CdS–TiO_2_/C NFs showed better photocatalytic activity due to their high surface area and beneficial electron transfer characteristics. It is believed that when the two semiconductors coupled, they had a useful influence on the charge separation and response of TiO_2_ to the visible light region. Here, CdS NPs separated electrons and holes, resulting in a better TiO_2_ photocatalytic performance. The synergetic effects of the NFs and the catalytic TiO_2_ and CdS NPs reduced the optical bandgap energy to 2.95 eV. C NFs also enhanced the photocatalytic reaction by adsorbing AB molecules on the surface. Figure 21 shows the catalytic performance enhancement by the addition of CdS and C to TiO_2_.

The reusability of CdS–TiO_2_/C NFs was studied by testing the photocatalytic performance of the same sample three times. H_2_ production was lower in the second and third cycles. This may be due to the decrease in the adsorption activity with the time of the catalysts.

### 8.8. CdS NPs/CdTiO_3_ NFs

CdS, as explained earlier, is an important photocatalyst and can be enhanced to overcome its issues by coupling with another wide bandgap semiconductor. Pant et al. have enhanced the photocatalytic performance of CdS NPs by coupling them with cadmium titanate (CdTiO_3_) [113]. CdTiO_3_ was chosen due to its extraordinary dielectric, sensing, and optical characteristics.

CdS NPs/CdTiO_3_ NFs were prepared by ES, followed by high-temperature air calcination. First, TTIP was mixed with AA for 10 min. Second, PVP and ethanol were added to the TTIP solution. Cadmium acetate (CdAc) and ammonium sulfide were added dropwise to the previous solution. The sol–gel was mixed for 12 h, and then the ES of the sol–gel was performed under 20 kV and at a 15 cm gap distance. The electrospun mat was vacuum dried at room temperature for 12 h and calcined in air at 600 °C for 3 h.

The characterization of the electrospun mats before and after heating revealed the fabrication of uniform cross-section NFs. The calcination resulted in the removal of PVP and the fabrication of CdS NPs (~8 nm) on the surface of CdTiO_3_ (~175 nm).

The prepared catalyst showed a lower emission intensity than CdTiO_3_. Hence, the catalyst reduced the recombination rate of the excited electrons and holes, ensuring better photocatalytic activity.

**Figure 22:**
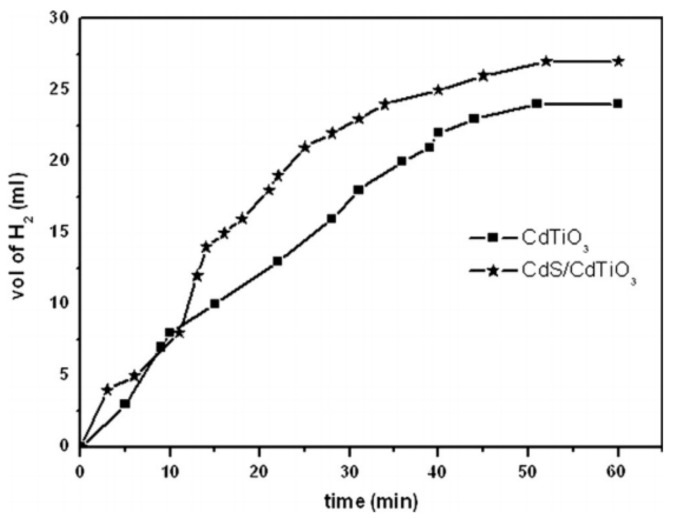
Photocatalytic performance of CdTiO_3_ NFs with and without CdS for AB hydrolysis reaction. Reprinted from reference [113] with permission from Elsevier.

CdS NPs/CdTiO_3_ NFs showed better photocatalytic activity under sunlight toward AB hydrolysis than that shown by CdTiO_3_ as shown in Figure 22. The generated H_2_ volume was ~27.5 and ~25 mL for the CdS NPs/CdTiO_3_ NFs and CdTiO_3_ NFs, respectively. The better photocatalytic activity was achieved due to the high degree of crystallinity of CdS NPs, which resulted in great synergetic effects for charge separation.

The catalyst was also studied for methylene blue degradation and showed high photocatalytic activity.

### 8.9. Zn–Fe-Doped TiO_2_ NFs

Zinc oxide (ZnO) and iron oxide (Fe_2_O_3_) were added to enhance the photocatalytic activity of TiO_2_ NFs [7]. The NFs were prepared by sol–gel, ES, and calcination processes. First, ES solutions containing TIIP, AA, ethanol, PVP, zinc acetate, and iron acetate were prepared; 20% ZnO and different amounts (0.25, 0.5, 1, and 2 wt%) of Fe_2_O_3_ were incorporated into the TiO_2_ NFs. Second, ES under 20 kV and at 15 cm was utilized to make polymeric NFs. Finally, after drying the NFs for a day at 20 °C, the prepared NFs were calcined in air at 700 °C for 1 h to make the desired catalysts.

**Figure 23:**
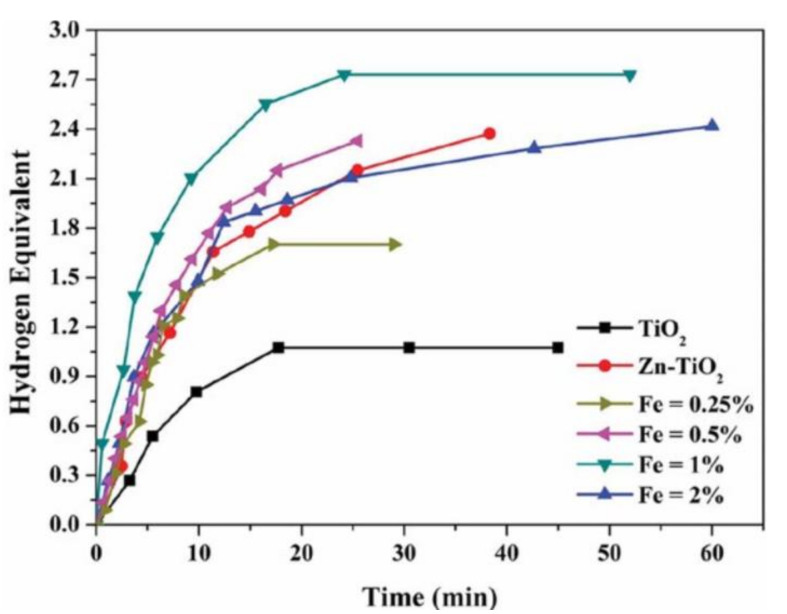
Photocatalytic performance of TiO_2_ NFs with and without Zn and Fe for AB hydrolysis reaction. Reprinted from reference [7] with permission from Taylor & Francis.

The photocatalytic activity of TiO_2_ toward AB hydrolysis was tested under visible light radiation using a mercury lamp. TiO_2_ catalytic activities were strongly enhanced by ZnO–Fe_2_O_3_-doping. It was found that pristine TiO_2_ NFs have the lowest catalytic activity, and 1 wt% Fe_2_O_3_–ZnO–TiO_2_ NFs have the highest activity among the utilized NFs as shown in Figure 23. Liu et al. have contended that since TiO_2_, ZnO, and Fe_2_O_3_ have different work functions and bandgap energies, the illumination of TiO_2_ results in the accumulation of the electrons and holes on the CBs and valance bands of Fe_2_O_3_ and ZnO, respectively [79]. Therefore, the results show that the surface of the fabricated NFs is active, which results in immediate AB hydrolysis upon reaching the active zone surrounding the NFs. Barakat et al. have showed that the H_2_ release increased when the temperature increased up to 35 °C; moreover, the rate decreased when the temperature was further increased to 40 °C. Finally, kinetic studies showed that the AB reaction using the NF catalysts is a zero-order reaction.

## 9. Cobalt NFs as a Catalytic Support for AB Hydrolysis Reaction

Since Co is a very promising catalytic metal, unsupported Co NFs and Co catalysts have also been prepared. As shown in Table 5, these Co catalysts are not supported on C or TiO2 NFs.

### 9.1. Co, Ni, and Cu Oxide NFs

Filiz and Figen have fabricated unsupported CoO, NiO, and CuO NF catalysts [17]. The authors studied different parameters, including solution concentration and ES parameters, on the fabricated NFs.

To prepare the catalytic NFs, first, 5 wt% PVA was dissolved in deionized water (DI) water. The PVA solution was stirred for 5 h at 80–90 °C and, further, for a day at room temperature to obtain a homogenous solution. Three different ES solutions were subsequently prepared by dissolving the metal acetates of Co, Ni, and Cu in the PVA solution, followed by stirring for 2 h at 90 °C. After that, ES was performed at different parameters (20–30 kV and a 7.5–15-cm gap distance). The fibrous mats were dried for 6 h at 110 °C to remove the water solvent. The dried NFs were treated with heat (nonisothermal heating: 1 °C/min up to 450 °C, followed by isothermal heating: waiting for 450 °C at 4 h, and finally, nonisothermal cooling to room temperature at 1 °C/min).

Among all tested parameters, a PVA NF mat with good morphology was obtained under a 5 wt% PVA solution, 25 kV, 1 mL/h, and 7.5 cm. Thus, 5 wt% of the metal precursors was added to the 5 wt% PVP solution to prepare the catalysts. After calcination, 30% of the original metal precursor weight remained with a different structure and crystallinity.

The characterizations of the NFs proved the fabrication of metal and metal oxide NFs. Even though the NiO NFs were the only fabricated NFs that contained some beads, they showed the highest surface area (11.26 m^2^/g) due to their minimal pore diameter and bumpy surface. The fabricated Co oxide NFs contained 27.2% CoO and 72.3% Co_3_O_4_, while the Cu oxide NFs contained 84% CuO and 16% Cu_4_O_3_.

**Figure 24:**
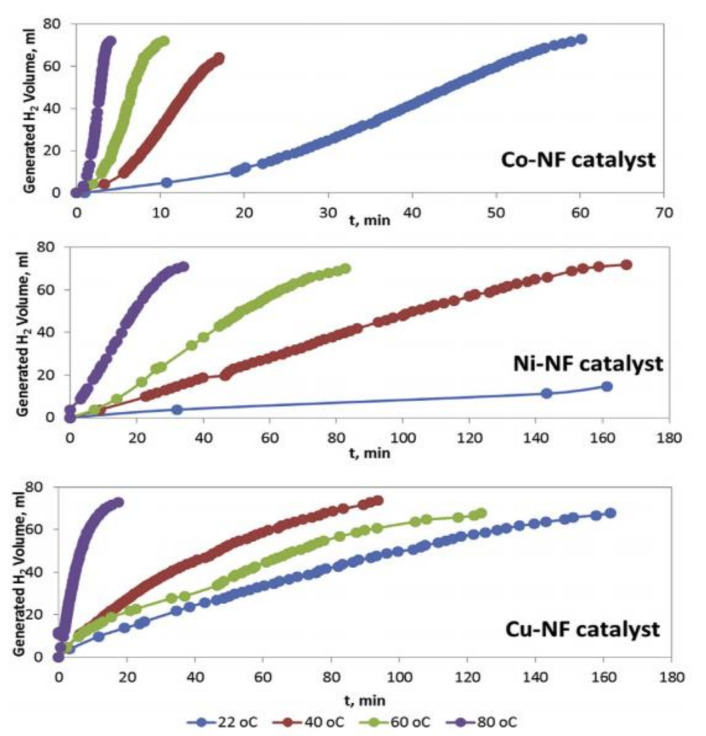
Catalytic performance of CoO, NiO, and CuO NFs at different temperatures in AB hydrolysis reaction. Reprinted from reference [17] with permission from Elsevier.

As shown in Figure 24, at a low temperature (22 °C), CoO and CuO NFs completely hydrolyzed AB. Higher reaction temperatures resulted in higher H_2_ production and lower release time. Co NFs showed the highest catalytic activity among all the tested catalysts.

Even though the catalytic activity depends greatly on the surface area, in this study, AB hydrolysis was greatly affected by the metal content. Since Ni and Cu NPs suffer from agglomeration under harsh conditions, Co showed the best activity toward AB hydrolysis. Kinetics studies showed that the reaction was independent of AB concentration (zero-order reaction).

The test for the recyclability of the catalyst was performed by using the same catalysts, which was carried out 10 times. All fabricated catalysts produced the theoretical maximum of H_2_ (3 mol). However, the release time increased with each consecutive cycle. The hydrogen generation rate was decreased by 43% for the fabricated Co NFs, 44% for Ni, and 82% for Cu after the tenth cycle, as compared to the rate in the first cycle.

### 9.2. Co–Mn–O NFs

Barakat et al. have fabricated manganese oxide-doped cobalt NFs (MnO-doped Co NFs) by ES, followed by calcination [13]. To prepare the catalyst, the cobalt acetate (CoAc) and manganese acetate (MnAc) were mixed with 10 wt% of the polyvinyl alcohol (PVA) aqueous solution. The ES of the prepared solution was further executed at 20 kV and a 15 cm gap distance. The NFs were dried and heat-treated in an Ar atmosphere at 850 °C to remove the PVP and convert MnAc and CoAc to MnO and Co, respectively. Theoretically, since Co and MnO have different physicochemical and atomic properties, they cannot form a solid solution alloy.

The characterizations of the heat-treated samples showed the fabrication of MnO-doped Co NFs with even beads. Usually, the calcination of metal acetates in an inert atmosphere, such as Ar, results in the generation of pure metals. However, MnAc was converted to MnO due to its high chemical activity. It is also very important to mention that in a previous study [13], the authors calcined CoAc/PVA and MnAc/PVA separately in an Ar atmosphere, and the acetates were converted to Co and MnO/Mn_3_O_4_. Thus, it can be concluded that the presence of Co catalyzes the formation of MnO.

**Figure 25:**
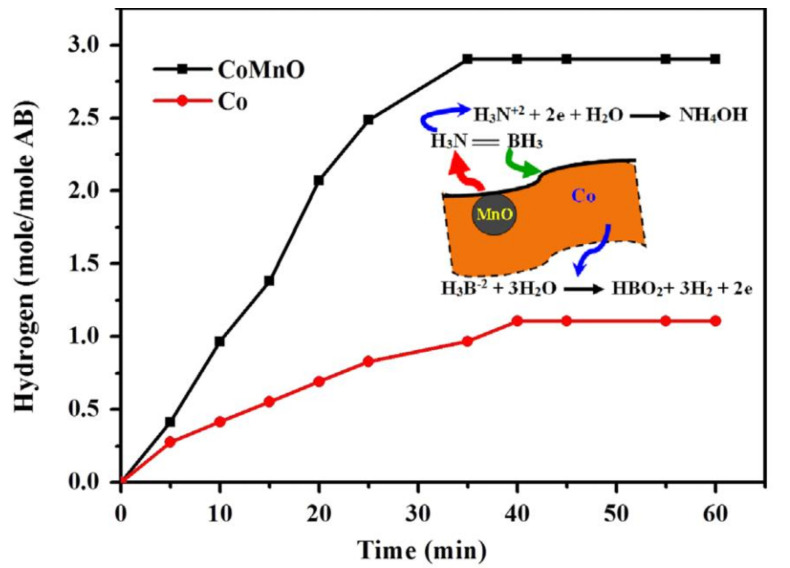
Catalytic performance of Co-MnO NFs and Co NPs in AB reaction. Reprinted from reference [13] with permission from Elsevier.

MnO-doped Co NFs showed a higher activity level than Co NPs for AB hydrolysis. The theoretical expected H_2_-generated moles were achieved in around 35 min, as shown in Figure 25. The author has proposed a mechanism for the hydrolysis reaction as follows:

MnO attacks the relatively positive group in AB (H_3_N^2+^), and the following reaction occurs on the MnO surface:

H_3_N^2+^ + 2 e^−^ + H_2_O → NH_4_OH (10)

Co oxidizes the acceptor group H_3_B^2^^−^, and the following reaction occurs on the Co surface:

H_3_B_2_^−^ + 2 H_2_O → H^+^ + BO^−^_2_+ 3 H_2_ +2 e^−^ (11)

The overall reaction matches the reported AB hydrolysis reaction, as shown in Equation (1).

### 9.3. Pd-Doped Co NFs

Alloying noble metals with first-row transition metals have unique and different magnetic, optical, electrical, and catalytic properties compared to monocatalysts. It is very important to control the size, morphology, and composition to obtain alloys with enhanced physical and chemical characteristics. The catalytic activity of the alloy can be enhanced even further by adding some C. Nasser fabricated Pd-doped Co NFs as bimetallic electrospun catalysts for normal and photohydrolysis of AB. Pd was chosen due to its high activity and affordability compared to Pt and Au. Co was chosen since it has the highest activity toward AB hydrolysis among non-noble metals [10].

The catalyst was prepared by mixing PVA and CoAc aqueous solutions very well. Furthermore, Pd NPs (<25 nm) were added to the solution and stirred for 5 h at 50 °C. The sol–gel solution was electrospun. The fabricated NFs were vacuum dried at 80 °C for a day to remove the water solvent. The dried sample was then calcined for 5 h at 700 °C (2.3 °C/min) under atmospheric pressure. It is noteworthy that the author used pure Pd NPs and not a palladium precursor. The characterization of the samples before and after calcination showed the fabrication of smooth and nonbeaded NFs. The samples contained Pd NPs (~26 nm) supported on face-centered cubic (FCC) Co NFs covered by a thin layer of graphite. Since the calcination process was vacuum operated, the pristine Pd did not change. Co and Pd contents in the final catalyst were in agreement with the initial content before ES was conducted (20% Pd and 80% Co). Due to the high melting point of Pd (1555 °C), Pd and Co did not melt with each other and form an alloy during calcination.

**Figure 26:**
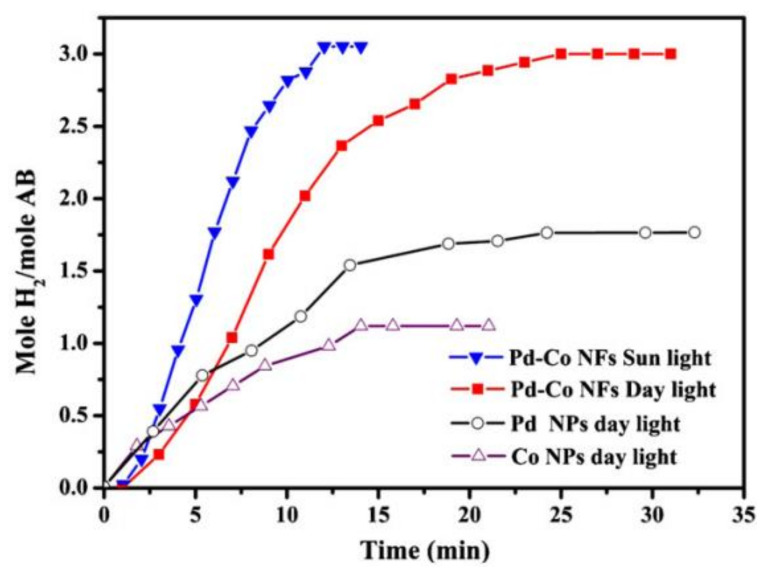
Catalytic performance of Pd, Co, and Pd-Co NFs in AB reaction. Reprinted from reference [10] with permission from Elsevier.

It is very important to note that usually, CoAc is converted to pristine Co rather than Co oxides when calcined in an inert atmosphere. Additionally, PVA graphitization is catalyzed more by the presence of Co than Pd–Co, and this explains the existence of a very thin graphite layer in the catalyst. The catalytic performance of Pd NPs/Co–C NFs was studied in sunlight and daylight, as shown in Figure 26, and compared with pristine Pd (>25 nm) and Co (>50 nm) NP performances. The prepared catalyst showed much higher activity than pristine Pd and Co NPs (~3 equivalent H_2_ moles compared to ~1.75 for Pd and ~1.1 for Co) in 20 min.

It is known that pristine metals such as Co and Ni NPs have no photocatalytic activity. Interestingly, Pd NPs/Co–C NFs showed high activity in daylight and sunlight. H_2_ was generated in a shorter time in the presence of sunlight. Hence, it is very clear that the catalyst possesses photocatalytic activity due to the bimetallic synergy effect. Since there was a possibility that the catalytic NFs would dissolve in water during the AB reaction, the author tested the solubility of the NFs in water. It is known that CoS and PdS are not soluble in water, so adding S^2−^ to a solution that contained Co or Pd ions would generate CoS and PdS precipitates, respectively. The reactions to produce CoS and PdS are straightforward, easy, and fast since the products are precipitates. The author tested the solubility of the prepared catalyst by adding a few drops of ammonium sulfide to the aqueous solution formed after the reaction. No precipitates were observed, indicating no Co or Pd in the solution. Hence, the catalyst was determined as chemically stable in water. The author believes that chemical stability is due to the existence of a thin layer of graphite shell.

The catalytic reusability was studied by testing the same catalyst seven times. No major catalytic performance change was observed. Moreover, the chemical stability of the NFs was maintained even after seven experiments.

## 10. Recommendation

Even though many valuable studies have been conducted to prepare efficient electrospun catalysts, there are still some research areas to be covered to move forward with AB hydrolysis using electrospun ceramic catalysts. Hence, this paper recommends carrying out the following investigations:

- Studying and improving the flow system operations of AB hydrolysis using electrospun ceramic catalysts;
- Studying and testing aluminum oxide NFs (Al_2_O_3_) as catalysts or catalyst supports;
- Conducting research to study the effect of trimetalic alloying;
- Studying and developing new electrospun biocatalysts;
- Modeling and simulation should be investigated to bring some conclusion that might help in saving energy and time to improve new catalysts.

## 11. Conclusions

The disadvantages of fossil fuels have encouraged many countries to search for alternatives. H_2_ is a great alternative due to its efficient power and cleanness. However, it is very hard to store and transport. Thus, H_2_ storage materials are used to generate it. Ammonia borane, referred to as AB in this paper, is a promising storage material. H_2_ can be generated from AB hydrolysis in the presence of an efficient catalyst. Electrospun ceramic catalysts are new kinds of catalysts that show very high catalytic and photocatalytic activities toward AB hydrolysis. This review discusses the published work in AB hydrolysis using electrospun ceramic catalysts.

## Figures and Tables

**Table 1:** A list of reviews published for various applications of electrospun NFs.

Review	Covered Application	Year	Reference
Electrospinning of polymeric nanofibers for drug delivery applications.	Drug delivery	2014	[55]
Advances in nanofibrous scaffolds for biomedical applications: From electrospinning to self-assembly.	Biomedical.	2014	[56]
Electrospinning and electrospraying techniques: Potential food-based applications.	Food Industry.	2014	[57]
Advances in three-dimensional nanofibrous macrostructures via electrospinning.	Tissue engineering. Energy harvesting and Storage, Filtration.	2014	[58]
Electrospinning for regenerative medicine: a review of the main topics.	Tissue engineering.	2014	[59]
Hierarchical electrospun nanofibers for energy harvesting, production, and environmental remediation.	Photovoltaics and photocatalysis. Hydrogen energy Harvesting, Fuel cells.	2014	[60]
Electrospinning of polymer nanofibers for tissue regeneration.	Medical.	2015	[61]
Fundamentals of electrospinning as a novel delivery vehicle for bioactive compounds in food nanotechnology.	Food technology.	2015	[62]
A review on electrospinning for membrane fabrication: Challenges and applications.	Water treatment.	2015	[63]
A comprehensive review summarizing the effect of electrospinning parameters and potential applications of nanofibers in biomedical and biotechnology.	Biomedical and biotechnology.	2015	[64]
Recent trends in electrospinning of polymer nanofibers and their applications in ultra-thin layer chromatography.	Chromatography.	2016	[65]
Melt electrospinning today: An opportune time for an emerging polymer process.	Energy, environment, filtration, and separation. Biomedical.	2016	[66]
A comprehensive review: electrospinning technique for fabrication and surface modification of membranes for water treatment application.	Water treatment.	2016	[67]
A review of polymer nanofibres by electrospinning and their application in oil-water separation for cleaning up marine oil spills.	Oil–water separation.	2016	[68]
Electrospinning: A versatile technique for making of 1D growth of nanostructured nanofibers and its applications: An experimental approach.	Energy conversion and storage. Environmental. Biomedical.	2017	[69]
Electrospinning: A novel nano-encapsulation approach for bioactive compounds.	Encapsulation of different types of bioactive compounds by biopolymer matrixes.	2017	[70]
Recent advances in multiaxial electrospinning for drug delivery.	Drug delivery.	2017	[71]
Electrospinning versus microfluidic spinning of functional fibers for biomedical applications.	Tissue engineering. Organ function regeneration. Drug delivery.	2017	[72]
Fibers for hearts: A critical review on electrospinning for cardiac tissue engineering.	Cardiac tissue engineering.	2017	[73]
Electrospun Nanofibers Membranes for Effective Air Filtration	Air Filtration.	2017	[74]
Electrospinning-based (bio)sensors for food and agricultural applications: A review.	Biosensor (Analysis of food/and agricultural products).	2017	[75]
Electrospinning in solid oxide fuel cells–A review.	Solid oxide fuel cells.	2017	[76]
Polymer-based composites by electrospinning: Preparation & functionalization with nanocarbons	Tissue engineering Chemical Biosensors Environmental remediation.	2018	[77]
An overview of electrospun nanofibers and their application in energy storage, sensors, and wearable/flexible electronics.	Wearable/flexible electronics.	2017	[78]
Recent advances in energy materials by electrospinning.	Energy-related devices.	2018	[79]
Non-precious nanostructured materials by electrospinning and their applications for oxygen reduction in polymer electrolyte membrane fuel cells.	Fuel cell (Oxygen reduction reaction in a fuel cell).	2018	[80]
Designing function-oriented artificial nanomaterials and membranes via electrospinning and electrospraying techniques.	Tissue engineering and medicine. Membrane filtration. Lithium battery.	2018	[81]
Electrospinning: An enabling nanotechnology platform for drug delivery and regenerative medicine.	Biomedical. Regenerative medicine.	2018	[82]
Emulsion electrospinning: Fundamentals, food applications, and prospects.	Food.	2018	[83]
Electrospinning and electrospray of bio-based and natural polymers for biomaterials development.	Food Industry. Enzyme immobilization. Tissue engineering. Drug delivery. Wound dressing.	2018	[84]
Comprehensive review on the electrospinning of starch polymer for biomedical applications.	Biomedical applications.	2018	[85]
Electrospinning tissue engineering and wound dressing scaffolds from polymer-titanium dioxide nanocomposites.	Tissue engineering. Wound dressing.	201	[86]
Electrospun nanofiber reinforced composites: a review.	Reinforced composites.	2018	[87]
ZnO-based ceramic nanofibers: Preparation, properties and applications	ZnO-based CNF applications.	2019	[88]
A review on electrospinning nanofibers in the field of microwave absorption	Microwave Absorption.	2020	[89]

**Table 2:** Catalytic data of C NF-supported catalysts for AB hydrolysis.

Catalyst	Catalytic Preparation Procedure	Catalytic Characterization Equipment	Reactor Type and Reaction Temperature (°C)	AB Concentration (mM)	TOF (mol H_2_/mol of Metal. min)	Reaction Order	Ea kJ/mol	Reference
NiCu NRs/C NFs	ES followed by calcination	SEM, FE-SEM, XRD, XPS, TGA and TEM-EDX	Batch 25, 30, 35, 40	13.33	-	-	28.9	[54]
Co-TiC NPs decorated C NFs	ES followed by calcination	SEM, FE-SEM/EDX, TEM, HR-TEM, TEM-EDX	Batch 25	100	32.18	Pseudo-1st with respect to the catalyst concentration	26.19	[9]
CoCr_7_C_3_- supported C NFs	ES followed by calcination	SEM, FE-SEM, XRD, TEM, HR-TEM, EDX, ICP-OES	Batch 25, 30, 40, 50	100	25.78	Pseudo-zero-order with respect to AB Pseudo-1st concerning the catalyst	24.2	[9]
NiCr NPs/C NFs	ES followed by calcination	SEM, FESEM, XRD, TEM, HR-TEM, TEM-EDX	Batch 25, 30, 35, 40, 45, 50	100	5.78	Pseudo-zero-order for AB Pseudo-1st to the catalyst	37.6	[6]

**Table 3:** TiO_2_ Modification techniques.

Technique	Purposes	Example	References
Doping with transition metals	Control the band gap of TiO_2_ that effectively helps to generate electron and hole pairs when using visible light instead of ultraviolet light. Formation of Schottky barrier at the metal and TiO_2_ interface that works as efficient electron trap to reduce the recombination rate of electrons/holes.	Ni_(0)_-TiO_2_/C NFs Cu_(0)_ NPs/TiO_2_ NFs	[14,108,109]
Loading a metal oxide	Reduction of recombination rate of electrons/holes.	CuO/TiO_2_	[8]
Surface modification by forming a composite system from combing two semiconductors	The synergistic effect can give better charge separation and chemical stability.	CdS–TiO_2_-doped C NFs	[110]

**Table 4:** Photocatalytic data of metal/metal oxide NP-supported TiO_2_ NFs for AB hydrolysis.

Catalyst	Catalytic Preparation Procedure	Catalytic Characterization Equipment	Reactor Type and Reaction Temperature (°C)	AB Concentration (mM)	Reaction Order	Ea kJ/mol	Reference
Ni-doped TiO_2_ NFs	ES followed by calcination	FE-SEM, HR-TEM, XRD, EDX, XPS, TGA, UV-visible spectrophotometer and photoluminescence (PL) spectroscopy	Batch Sunlight	8.4	-	-	[103]
Ni(0)-doped TiO_2_/C NFs	ES followed by calcination	XRD, TEM, TEM-EDX	Batch Sunlight	13.33	-	-	[15]
CuO/TiO_2_ NFs	ES followed by calcination	SEM, FE-SEM, EDX, XRD, TEM, HR-TEM, XPS	Batch Sunlight Day light	13.33	-	-	[16]
Cu(0) NPs/TiO_2_ NFs	ES followed by Hydrothermal	SEM, FE-SEM, XRD, TEM, HR-TEM, EDX, XPS	Batch Sunlight	13.33	-	-	[14]
Cu-doped TiO_2_/C NFs	ES followed by calcination	XRD, FE-SEM, TEM, HR-TEM, EDX	Batch Sunlight	13.33	-	-	[111]
Cu(0)/S-doped TiO_2_ NPs/C NFs	ES followed by calcination	FE-SEM, EDX, TEM, HR-TEM, XRD	Batch Sunlight by Mercury lamp	13.33	-	-	[8]
CdS–TiO_2_-doped C NFs	ES followed by calcination	FE-SEM, TEM, TEM, HR-EDX, XRD, TGA	Batch Sunlight by Mercury lamp	13.33	-	-	[112]
CdS NPs/CdTiO_3_ NFs	ES followed by calcination	FE-SEM, EDS, TEM, HR-TEM, XRD	Batch Sunlight	13.33	-	-	[113]
Zn–Fe-doped TiO_2_ NFs	ES followed by calcination	SEM, FE-SEM, EDX, XRD, TEM, HR-TEM	Batch Visible light irradiation using by Mercury lamp	13.33	Pseudo-zero-order for AB	Negative value	[16]

**Table 5:** Catalytic data of Co NFs for AB hydrolysis.

Catalyst	Catalytic Preparation Procedure	Catalytic Characterization Equipment	Reactor Type and Reaction Temperature (°C)	AB Concentration (mM)	Reaction Order	Ea kJ/mol	Reference
CoO NFs	ES followed by calcination	XRD, FT-IR, BET, SEM	Batch 22–80	13.33	zero-order reaction	35.4	[17]
Co-Mn-O NFs	ES followed by calcination	XRD, SEM, TEM, TGA	Batch	13.33	-	-	[13]
Pd-doped Co NFs	ES followed by calcination	SEM, FE-SEM, EDX, XRD, TGA, TEM, HR-TEM, Raman	Batch Sunlight	13.33	-	-	[10]

## Data Availability

Not Applicable.
